# Preparation and validation of the instrument “QualiAPS digital—Brazil” for assessing digital health care in primary health care: a required tool

**DOI:** 10.3389/fpubh.2024.1304148

**Published:** 2024-07-16

**Authors:** Renan Cabral de Figueirêdo, Ísis de Siqueira Silva, Aguinaldo José de Araújo, Cícera Renata Diniz Vieira Silva, Cláudia Santos Martiniano, Ewerton William Gomes Brito, Pedro Bezerra Xavier, Severina Alice da Costa Uchôa

**Affiliations:** ^1^Department of Public Health, Federal University of Rio Grande do Norte, Natal, Brazil; ^2^Department of Dentistry, Federal University of Rio Grande do Norte, Natal, Brazil; ^3^Technical School of Health of Cajazeiras, Federal University of Campina Grande, Cajazeiras, Brazil; ^4^Department of Nursing, State University of Paraíba, Campina Grande, Brazil; ^5^Health Sciences Center, Federal University of Rio Grande do Norte, Natal, Brazil

**Keywords:** telemedicine, digital health, health assessment, validation study, primary health care

## Abstract

**Introduction:**

The use of Information and Communication Technologies in the field of health is increasing across the world, demarcating the field of digital health. The goal of this study is to formulate and validate a matrix of indicators, design assessment scripts and indicate data collection techniques for assessing the quality of digital health care in Brazilian Primary Health Care (PHC).

**Methodology:**

This is a validation study divided into three phases: preparation of the instrument, analysis of validity and pilot study. The instrument was prepared based on the PHC assessment model from a literature review; the analysis of validity used the Delphi technique associated with the nominal group and the evidence from the literature reference. In the pilot study, audio-recorded interviews were conducted with strategic primary care actors.

**Results:**

The matrix of indicators “QualiAPS Digital—Brazil” introduces a set of 37 indicators, distributed into three distinct components and their respective dimensions. The component “Structure” includes the dimension “Resources”; the component “Processes” includes the dimensions “Technical,” “Organizational” and “Relational”; and the component “Results” includes the dimensions “Short-Term Results” and “Medium-Term Results.” The general values obtained for CVI and IRR were 0.89 and 1.00; respectively. Therefore, it was possible to design assessment scripts and indicate qualitative data collection techniques for assessing digital health in Brazilian PHC.

**Conclusion:**

The instrument presented was validated regarding its relevance, content and theoretical support to evaluate the quality of digital health care, supporting decision-making by managers and health professionals in the search for improving remote primary care provided to the population.

## 1 Introduction

Digital transformation within the context of globalization stands as an irreversible reality, exerting significant influence on national health services through the increasingly pervasive application of Information and Communication Technologies (ICTs). This phenomenon is particularly pronounced as health systems globally contend with financial limitations and escalating demands for elevated care quality (1, 2). The uptake of ICTs in healthcare surged notably throughout the 20th century, manifesting in diverse domains and fostering improved communication and information dissemination. These technologies effectively transcend traditional constraints of temporal and spatial limitations, facilitating the more effective and timely delivery of healthcare services. Remote consultations, telemedicine, and electronic health records represent key applications of ICTs, thereby enhancing patient outcomes and streamlining operational processes. As ICTs continue to advance, they present innovative solutions to longstanding challenges, ensuring that healthcare systems are equipped to fulfill rising expectations regarding accessibility, efficiency, and care quality (3–5).

The term “digital health” was introduced by the World Health Organization (WHO) as a broad umbrella covering the use of electronic and mobile technologies (ICTs—a set of technological resources integrated through hardware, software, and telecommunications) to support and promote remote clinical health care, patient and professional education, public health, and health administration (1, 4, 5). It covers strategies known as telemedicine, telehealth, telecare, teleconsultation, e-Health, videoconsultation, virtual health, remote consultation, among others (6). Established as a priority since 2005, the digital transformation has a significant impact on the field of health, providing the conditions for redefining the health care model, making it more integrated, participatory, and personalized (7, 8).

This transformation ranges from using the telephone to respond to patient questions, to modern video capabilities on smartphones and text messaging through mobile app tools and social media. These strategies are advantageous for extending healthcare access and resources, especially in remote or underserved areas where healthcare teams are scarce. They diminish patient travel requirements, simplify appointment scheduling, and enable the efficient renewal of medical prescriptions. Moreover, these changes influence the doctor-patient dynamic and may foster a heightened focus on self-care (4, 9). The integration of technologies into the healthcare sector necessitates advancements in assessing care facilitated through digital resources (10). Evaluating the impact of digital health on healthcare delivery can enhance care quality by identifying opportunities to leverage these technologies effectively and understanding their limitations. This process empowers healthcare providers to optimize their utilization of digital tools, ensuring they complement traditional care methods efficiently. By doing so, healthcare organizations can aim for greater efficiency and patient-centeredness, ultimately enhancing outcomes and accessibility for patients (11, 12).

In the context of Brazil, in 2021, the Ministry of Health (MS, as per its Portuguese acronym) started the process of strengthening the use of digital health and defined the priorities for this area, with the publication of the Digital Health Strategy for Brazil 2020–2028 (ESD28, as per its Portuguese acronym). In addition to the publication of this guiding document, MS has encouraged the adoption of technologies through the Program for Supporting Computerization and Qualification of Primary Health Care Data (*Informatiza APS*), with financial incentives to municipalities, use of information systems (such as e-SUS/APS) and adoption of the Electronic Citizen Record. Within ESD28, MS considers it important to establish minimum quality standards and a continuing assessment of the level of digital maturity of public and private facilities (13, 14).

In December 2022, digital health is regulated by the Presidency of the Republic through Law 14,510, supported by previous legislation that deals with the civil landmark for internet use, data protection and the exercise of health professions. In addition, municipalities have autonomy in adhering to and developing digital health (15, 16). In 2024, as part of the ESD, the Ministry of Health implemented the SUS Digital Program with the purpose of expanding access to health actions and services for the population, aiming for comprehensive and effective health care. This digital transformation should cover all areas of health, including comprehensive care, health surveillance, training and continuing education for professionals, management of the SUS in all its instances, including primary health care (PHC), as well as planning, monitoring, evaluation, research, development, and innovation in health, without excluding other areas (17).

However, as far as evaluation is concerned, consolidated instruments based on scientifically validated evaluation models are not yet available. Aligned with the perspective of developing evaluative research that agrees with the propositions of the SUS Digital Program, the study proposed here is part of the project “Assessing the quality of telemedicine in Primary Health Care in times of crisis due to COVID-19.” The scoping review, included in this project, carried out by Silva et al. (6), highlighted eight frameworks for assessing digital health validated in different countries. The model proposed by Christiansen et al. (18) highlights the importance of cultural context, user needs and leadership. The model by Finch et al. (19) emphasizes adaptation to health needs, cognitive involvement of professionals and monitoring. Glasgow et al. (20) focus on population access, technology effectiveness, adoption, and sustainability. Khoja et al. (21) emphasize the results of services and technologies, as well as ethical, political and improvement aspects. Kidholm et al. (22) highlight financial aspects, purpose, maturity of the service and patient perception. Kidholm et al. (23) underline quality, convenience, technical difficulties, and health effects. Nepal et al. (24) drive the evaluation towards areas of application in health, types of tools and devices, communication technologies and socio-economic factors. Shaw (25, 26) includes clinical, human, interpersonal, educational, administrative, and technical aspects.

Although each of the models brings important domains to be incorporated into the evaluation of digital health, they do not express the interrelationship between the domains present in each model and their influence on the results. Although they unveil the effects of the use of technologies, they do not consider the impacts on strengthening the health systems where they are implemented, which is the issue of interest in this work. The option was to draw up a model considering the context of Brazilian digital health, especially based on Donabedian’s systemic approach (26, 27), comprising the interconnection of structure (technological infrastructure, human resources, policies), the process of implementing and using technologies and its influence on the results of the use of technologies and impacts on the essential attributes of PHC, as proposed by Starfield (28). For instance, a robust technological infrastructure and well-trained human resources ensure the efficient integration of technologies into daily workflows, while clear policies and regulations guide their safe and effective use. This process, in turn, impacts outcomes by determining the efficiency and effectiveness of technology utilization. Proper implementation and continuous training result in more accurate diagnoses, more effective treatments, and higher patient satisfaction. Ultimately, these outcomes feed back into the structure and process, justifying further investments in technological infrastructure, training, and policy enhancements. Positive outcomes and feedback facilitate continuous adjustments and improvements, creating a cycle of enhanced healthcare quality through the optimal use of digital technologies in primary healthcare (PHC).

The relationship between the essential attributes of PHC and the adoption of digital health is fundamental in terms of understanding how technologies can strengthen health services. Firstly, digital health can improve first contact access by allowing patients to get in touch with health professionals quickly and conveniently, through platforms such as mobile apps and telemedicine. In addition, longitudinality, one of the pillars of PHC, can be improved with the implementation of digital technologies that guarantee access to medical information and patient records over time, promoting continuity of care (6, 7). As for integrality, the use of digital tools makes it possible to promote a more holistic approach to patient care. The coordination of care, family and community orientation and the cultural competence of managers, professionals and users also need to be considered when evaluating care by digital means (28). Although digital health has a broad spectrum, the focus of this work is the application of ICTs in health care, which can be understood in the dimensions proposed by Donabedian, such as technical, organizational, and relational (26, 27).

Based on this set of perspectives, the “Model for Assessing Digital Health in PHC in Brazil” (29) was designed, available in the Supplementary material of this article. Under the perspective of this model, the aim of this study is to formulate and validate a matrix of indicators, design assessment scripts and indicate data collection techniques for assessing the quality of digital health care in Brazilian PHC. The development of instruments to evaluate digital health in PHC in Brazil, considering its digital health context, its scope, diversity, and social inequalities, is extremely important given the growing role of digital technologies in the provision of health care. These instruments enable a systematic and objective evaluation of the impact of digital technologies on the quality, accessibility and effectiveness of health services provided in PHC.

## 2 Digital health context

With the outbreak of the COVID-19 pandemic in 2020, social, economic and health impacts emerged, which required rapid adaptations of health systems, so that care provided to people could be continued. At that time, the WHO highlighted Primary Health Care (PHC) as a key point, guiding the reorganization and expansion of health services in the sense of combating the pandemic and maintaining other services. It was in this context that the use of ICTs gained great prominence with their prodigious effect in terms of fulfilling this goal, although it is not known to what extent this technology has been incorporated into the health system to guarantee its sustainability (6, 30). During this process of change, national and international agencies published documents with evidence-based arguments about the importance of using and implementing digital health to expand access and optimize the provision of health services. These regulations opened space for movement between health bodies, with consonances and specificities regarding the challenges for implementing digital health care (31).

Having gone through this period of accelerated growth of digital health in recent years, its fundamental role in achieving universal health coverage and supporting efforts to make healthcare more efficient, accessible and effective is now clearly recognized (32). Digital health technologies offer tangible opportunities to meet the challenges of the health system and improve the reach and quality of care and services. Digital health has been the subject of research and can be used from highly complex health services to home-based primary care (33, 34). Its use, with proper management, guidance and coordination, facilitates direct communication with users, expands coverage and access to information, care and self-care. In addition, health professionals have access to clinical protocols and records more rapidly (35, 36).

When integrated into PHC, the preferred gateway and foundation of health systems, digital health plays a significant role in strengthening public health systems, aligning them with the Sustainable Development Goals (37). The strengthening of a universal system is closely linked to the quality of access to health and digital health provides excellent contributions to the organization of services and health actions in PHC and the Brazilian Unified Health System (SUS), considering the continental dimension of the country and remote communities with little access to health professionals and services (38).

PHC has used digital tools to expand access to health, digitize medical records, promote continuity of care, carry out teleconsultations, monitor vital signs, among others (39, 40). According to the WHO, digital health is a broad, economical and safe field for the application of Information and Communication Technologies (ICTs), its scope includes, but is not limited to telemedicine, telehealth, platforms, health information systems, electronic records; mobile health (mHealth); emerging technologies, such as “big data,” genomics and artificial intelligence (1).

Nevertheless, the advances in the application of technologies in the field of health reveal another face, already known from globalization, which is materialized in the digital divide. The implementation of digital health in universal health systems must be linked to the concepts of equity of access and sustainability (41). Furthermore, there are notable disparities when it comes to digital infrastructure, according to the International Telecommunications Union (ITU), Internet access is available to only 53.6% of the global population, with significant variations between different nations (42).

When considering equity, it is fundamental to ensure that technologies are accessible and fair to everyone, regardless of their location, socioeconomic status, or ability to access resources. This means eliminating barriers to access and ensuring that the benefits of digital health extend to all members of society, contributing to a more inclusive and fair health system. In order to promote equity and guarantee the benefits provided by the digital transformation of health, certain conditions are identified, such as access to infrastructure and/or connectivity, digital literacy skills and motivation to use digital technologies (43).

Still, other strategic measures are necessary for the sustainability of a strengthened PHC, such as guaranteeing resources, actions by multiple sectors/agents and viable public policies for the most unique territories, offering continuous and longitudinal care, with guaranteed timely and quality access (44). For Hadjiat (45), it is appropriate to thoroughly analyze existing digital alternatives and create new approaches with the specific aim of combating health disparities; implementing solutions in a careful and reflective manner, aligned with demands; and considering economic, social and development aspects, thus contributing to the sustainability of actions.

Digital health has seen varying levels of growth across the world, which can be attributed to socioeconomic factors, such as income, education, poverty, and digital infrastructure. Higher-income countries, such as the United States and Japan, have greater access to digital health technologies. On the other hand, low-income countries, such as Bangladesh and Ethiopia, face challenges in implementing these technologies due to limited resources and infrastructure. Middle-income countries, for example, Brazil and India, are in an intermediate position (46). The Brazilian government’s initiatives to encourage the adoption of digital health in PHC were influenced by the experiences of countries with universal health systems, such as Canada in the use of Canada Health Infoway to successfully develop interoperable electronic records in PHC and Australia with the My Health Record in the expansion of electronic medical records (47–49).

## 3 Methodology

### 3.1 Context and study design

**Figure 1:**
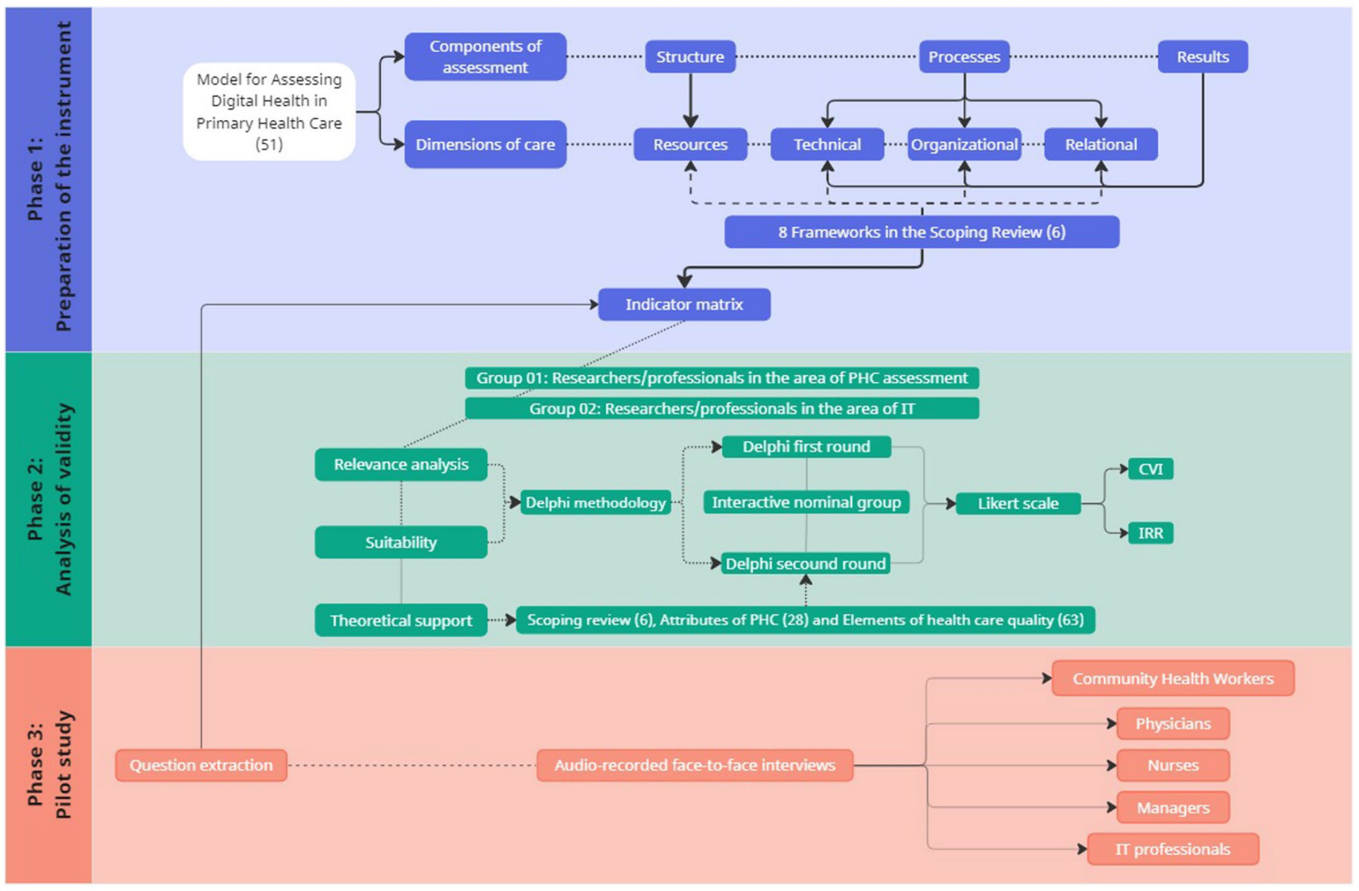
Flowchart of the methodological steps.

It is a methodological study whose goal is to develop relevant, adequate and accurate instrument that can be used by other researchers and the general population. It seeks to develop validation, assess tools and improve a technology (50). It was developed between January 2022 and June 2023, involving three phases: Preparation of the instrument, analysis of validity (relevance, suitability, and theoretical support) and pilot study, as displayed in Figure 1.

### 3.2 Phase 1: preparation of the instrument

The matrix of instrument indicators, called “QualiAPS Digital—Brazil,” was prepared based on the “Model for Assessing Digital Health in Primary Health Care” proposed by Silva and Uchoa (51), considering the components of assessment quality, structure (resources), processes (activities) and results, as well as the technical, relational and organizational dimensions of care (26, 27).

Once it had been prepared, the matrix of indicators was sent via e-mail in Google doc format to seven researchers who were collaborating on the project, with suggestions for changes, deletions, or additions of indicators. The suggestions were debated in an online Discussion Group (52), held in July 2022, and those that reached consensus were incorporated. At that time, data collection techniques and informants were also agreed upon, resulting in version 1 of the matrix, which was submitted for validation by experts in Digital Health.

### 3.3 Phase 2: analysis of validity

This phase included the analysis of relevance, suitability (content) and theoretical support of each potential indicator of “QualiAPS Digital—Brazil.” Relevance indicates how important the indicator is in the assessment conducted by the judges; suitability, how well it is able to measure the reality being assessed; and theoretical support refers to the literature on what evidence supports the indicator (53). The relevance and adequacy of the indicators were subjected to the judgment of experts, whose data was collected using the Delphi technique (54) through the application of a Likert scale.

Experts from two groups were considered, one of researchers/professionals in the area of PHC assessment and the other of researchers/professionals in the area of Information Technology (IT). The expertise criterion for both groups was experience in digital health in the academic environment, in telemedicine/telehealth/digital health centers and/or in health services, confirmed by publications (articles and surveys) in curricula, a record of technical experience in institutions, preferably Brazilian institutions with technical-scientific recognition. The sample was typified as an intentional “snowball sample” by successive indication (55) and was recruited by telephone, WhatsApp^®^ and/or e-mail with a presentation of the goals, the Delphi methodology and the Free and Informed Consent Form.

The Delphi panel was developed online in two rounds, for each of which a Likert scale (1 to 4) was created for relevance and suitability (content) of the potential indicators, considering: 1 = Item not relevant and not suitable; 2 = Item needs major review to be relevant and suitable; 3 = Item needs minor review to be relevant and suitable; and 4 = Item relevant and suitable. After each item, spaces were provided for possible qualitative suggestions.

In order to carry out the Delphi first round related to the validation of “QualiAPS Digital—Brazil,” 25 invitations were sent electronically to experts in Digital Health. When the invitations were not responded to in a certain period, they were sent again, resulting in a final sample of 17 respondents for the Delphi first round. The data was collected between July and October 2022, thus generating the first database available in the Supplementary material of this article. The Delphi second round of the validation was carried out between December 2022 and February 2023, following the same format as the first. It was sent to the 17 judges in the sample, with 15 respondents (abstention rate between rounds of less than 12%). The second database can be consulted in the Supplementary material of this article.

### 3.4 Data analysis

The databases generated from the Delphi rounds were organized in .xlsx format in Excel 2020 software and statistically analyzed in SPSS, version 25.0. The analysis used the Content Validity Index (CVI) for each indicator and the Inter-Rater Reliability or Concordance Index (IRR) for the proposed dimensions. In order to calculate the CVI index for each item in the instrument, the responses 3 and 4 of the experts were added together and the result divided by the total number of responses obtained for the item (as shown below). The overall CVI of the instrument was calculated by adding up all the CVI indexes calculated separately, divided by the number of items. An acceptable content validity index must be at least 0.80 (56).

$CVI=\frac{\text{number}\;\text{of}\;\text{equivalent}\;\text{responses}\;\left(3\;\text{and}\;4\right)\;\text{from}\;\text{experts}}{\text{total}\;\text{number}\;\text{of}\;\text{responses}\;\text{for}\;\text{the}\;\text{item}}$

Regarding the IRR index, which is designed to assess the extent to which the judges are reliable, the researcher counted how many items were rated 3 or 4 and 1 or 2. For each dimension of the instrument, the number of items that obtained at least 80% agreement among the raters was divided by the total number of items in each dimension (56). As show below:

$IRR=\frac{\begin{array}{l} \text{number}\;\text{of}\;\text{items}\;\text{with}\;\text{at}\;\text{least}\;80\%\text{agreement}\;\\ \text{between}\;\text{raters}\;\left(3\;\text{and}\;4\;\text{or}\;2\;\text{and}\;1\right)\;\text{per}\;\text{dimension} \end{array}}{\text{total}\;\text{number}\;\text{of}\;\text{items}\;\text{in}\;\text{each}\;\text{dimension}}$ Between the two Delphi rounds, an interactive and remote nominal group (57) was held, mediated by the Google Meet tool, in order to discuss the qualitative suggestions resulting from the opinions of the judges in the Delphi first round.

Theoretical support was assessed after relevance and suitability and based on the level of evidence of the literature reference that supports the recommendation for each indicator related to digital health, supported by the Scoping Review (6), particularly in the 08 frameworks selected for the preparation of the assessment model (51). The indicators were checked for consistency in relation to the attributes of PHC: access, longitudinality, integrality, care coordination, cultural competence and family and community orientation (28); and the elements of health care quality: equity, safety, timeliness of care, effectiveness, efficiency, integration of care and people-centered care (58).

### 3.5 Phase 3: pilot study

The pilot study took place between April and May 2023, in the municipality of Campina Grande/Paraíba, which is not part of the research project, with similar characteristics of PHC services in relation to the case where the instrument will be applied. In order to accomplish it, based on validated matrix, guiding questions were extracted from the indicators in such a way as to fully cover them, distributing them, initially, in four collection scripts for different professions involved in remote care: one for PHC physicians and nurses, one for Community Health Workers (CHWs), one for PHC managers and one for professionals involved in Information Technology (IT) also linked to PHC.

With the aim of testing and adapting the prepared scripts, the pilot was carried out with at least one representative from each category. The sample was intentional and had the criteria of being a PHC professional and having used some form of information and communication technology in the health care network during the COVID-19 pandemic. The used techniques were audio-recorded face-to-face interviews, guided by questions extracted based on indicators from “QualiAPS Digital—Brazil.”

The instrument intended for physicians and nurses, tested with one physician, contained 63 questions (12 open and 51 closed) covering indicators in all the dimensions of the instrument (“Resource,” “Technical,” “Organizational,” “Relational,” “Short-Term Results” and “Medium-Term Results”). The script intended for CHWs was tested with two representatives of the category, possessing 70 questions (13 open and 57 closed) covering indicators in all the dimensions of the instrument.

The instrument aimed at managers was tested with a professional with a degree in nursing, who held a position in municipal management at that time. The instrument contained 41 questions (12 open and 29 closed) relating to indicators in the dimensions “Resource,” “Technical,” “Organizational,” “Short-Term Results” and “Medium-Term Results.” And finally, the script intended at IT professionals, tested with one representative of the category, which initially contained 10 questions (two open and eight closed) distributed in the dimensions “Resources,” “Technical,” “Organizational,” “Short-Term Results” and “Medium-Term Results.” The instruments used in the pilot study are available in the Supplementary material of this article.

The interviews were transcribed in full, with the help of Transkriptor^®^ software, where adjustments were also made by the researchers. Based on the data obtained from the transcriptions, the authors improved the initial scripts, refining the pre-defined questions and those that emerged in the interviews, defining the target audience and the respective collection techniques.

A summary of the methodological steps can be seen in Figure 1.

### 3.6 Ethical aspects

The project was approved by the Research Ethics Committee of the Onofre Lopes University Hospital of the Federal University of Rio Grande do Norte under CAAE no 48655521.9.0000 and protocol number 4.859.682.

## 4 Results

### 4.1 Phase 1: preparation of the instrument

The initial version of the matrix of potential indicators of the instrument “QualiAPS Digital—Brazil” for assessing the quality of digital health care in PHC contains three components (Structure, Processes and Results) and five dimensions. The component “Structure” comprises the dimension “Resources” (R), with 14 indicators; the component “Processes” has three dimensions: Technical (T), with 04 indicators; Organizational (O), with six; and Relational (RE), with three. Finally, there is the component “Results,” which includes the dimensions “Short-Term Results” (ST), with six indicators; and “Medium-Term Results” (MT), with four, totaling 37 indicators, as well as the respective collection techniques and informants. The indicators are marked by the acronym indicating the respective dimensions above and followed by the sequential numerical order for each dimension. This version of the matrix is available in the Supplementary material of this article.

### 4.2 Phase 2: analysis of validity

The characterization of the sample is shown in Table 1.

**Table 1:** Characterization of the sample.

	*n* (%)	Gender	Mean age	Training	Degree	Experience in Digital Health
Experts in PHC assessment	10 (58.82%)	male (3) female (7)	41.1 years (maximum 52 and minimum 33)	Nursing (8) Pharmacy (1) Physiotherapy (1)	Doctorate (8) Master (2)	10
Expert professionals in IT	7 (41.18%)	male (7)	41.86 years (maximum 54 and minimum 35)	Computer engineering (3) Electrical engineering (2) Physical and technological engineering (1) Computer science (1)	Doctorate (7)	7

Source: Research data (2023).

#### 4.2.1 Delphi first round

In the Delphi first round to validate the proposed matrix, of the 37 indicators shown, the corresponding R3, R6, R8, R10 and R13, located in the component “Structure” and dimension “Resources,” obtained a Content Validity Index (CVI) insufficient for validation (CVI < 0.80), as displayed in Table 2.

**Table 2:** Validation of “QualiAPS Digital—Brazil.”

COMPONENT/DIMENSION	INDICATOR	DELPHI 1		INDICATOR	DELPHI 2		REALIGNMENT OF INDICATORS
		CVI	IRR		CVI	IRR	
STRUCTURE/ RESOURCE (R) 14 INDICATORS	R1	0.82	0.64	–	–	1.00	R1
	R2	0.88		–	–		R2
	R3	0.76		I II III	1.00 1.00 1.00		R3 R4 R5
	R4	0.88		–	–		R6
	R5	0.88		Deleted (similar to III)			–
	R6	0.76		IV	1.00		R7
	R7	0.82		–	–		R8
	R8	0.71		V VI (transferred to O3)	1.00 1.00		R9 –
	R9	0.82		–	–		R10
	R10	0.71		VII	1.00		R11
	R11	0.88		–	–		R12
	R12	0.82		–	–		R13
	R13	0.71		Deleted for having been covered			–
	R14	0.88		–	–		R14
PROCESSES/ TECHNICAL (T) 04 INDICATORS	T1	0.82	1.00	–	–	1.00	T1
	T2	0.94		–	–		T2
	T3	0.88		–	–		T3
	T4	0.82		–	–		T4
PROCESSES/ ORGANIZATIONAL (O) 06 INDICATORS	O1	0.88	1.00	–	–	1.00	O1
	O2	0.88		–	–		O2
	O3	0.88		Wording replaced by VI			O3
	O4	0.88		–	–		O4
	O5	0.88		–	–		05
	O6	0.82		–	–		O6
PROCESSES/ RELATIONAL (RE) 03 INDICATORS	RE1	0.94	1.00	–	–	1.00	RE1
	RE2	0.94		–	–		RE2
	RE3	0.82		–	–		RE3
RESULTS/ SHORT-TERM RESULTS (ST) 06 INDICATORS	ST1	0.88	1.00	–	–	1.00	ST1
	ST2	0.82		–	–		ST2
	ST3	0.82		–	–		ST3
	ST4	0.88		–	–		ST4
	ST5	0.82		–	–		ST5
	ST6	0.88		–	–		ST6
RESULTS/ MEDIUM-TERM RESULTS (MT) 04 INDICATORS	MT1	0.88	1.00	–	–	1.00	MT1
	MT2	0.82		–	–		MT2
	MT3	0.82		–	–		MT3
	MT4	0.88		–	–		MT4
OVERALL	37	0.85	0.94	–	0.89	1.00	37

Source: Research data (2023).

Table 2 shows the indexes obtained in the two Delphi rounds related to the validation and realignment of the indicators:

The indicators are named after their respective dimensions and followed by a sequential numerical order.

With this set of CVIs below those recommended for validation, the result was a low Inter-Rater Reliability or Concordance Index (IRR = 0.64) for this dimension. These indexes are not suitable according to the literature adopted in the methodology (56). Therefore, for non-validated indicators, the qualitative suggestions were then summarized and discussed in the Nominal Group (57), which brought together two authors and four judges. After this process, these indicators were readjusted, thus giving rise to another seven indicators, as highlighted in Table 3.

**Table 3:** Readjustment of non-validated indicators.

Non-validated indicators in the Delphi first round (CVI < 0.80)	Summary of the judges’ suggestions^*^	New indicators resulting from the Nominal Group
R3 (Number and categories of professionals from the Family Health Teams (e-FHS and e-PC) who develop or have developed digital health actions in the unit/household/community)	Separate number and categories of professionals from the same indicator	I (Number of Family Health Teams (e-FH and e-PC) that use/used remote care actions in the unit/household/community). II (Number of professionals who use/used remote care in the unit/household/community). III (Categories of professionals (health professionals, IT technicians, coordinators) involved in digital health actions in the unit, district or central level)
R6 (Geographical accessibility [possibility of access] and adjustment of the physical spaces of the Family Health Units (FHU) for multiple demands, face-to-face/remote, for COVID-19 and non-COVID-19 cases)	Remove geographic accessibility Do not relate to COVID-19. Consider virtual accessibility	IV [Adjustment of the physical and technological infrastructure of health units for the multiple demands (face-to-face and remote)]
R8 (Availability and quality of the internet, connectivity [computers with access to the internet network], and integration between systems [internet of things, such as sensors, intelligent monitoring that can be viewed on several devices at the same time])	Separate internet availability and quality from systems integration. Clarify the systems	V (Availability and quality of the internet in Health Units). VI (Interoperability between information systems (e-Sus and municipal systems) and devices/systems used for remote care)
R10 (Quality of information systems, management of the received data and interface with users of these technologies [health professionals and users])	Confusion of concepts Define the parameters used for quality	VII (Quality of the system [e-SUS/PeC or systems designed by the municipal management itself]). Perception of system response time; Robustness; Usability; Availability of tutorials/manuals; Functionality for remote care; Issuing reports
R13 (Information System including digital health actions)	Indicator considered with the others	DELETED

Source: Research data (2023). ^*^For more details, see Supplementary material of this article.

#### 4.2.2 Delphi second round

With the Delphi second round, the seven new indicators obtained CVI indexes of 1.00, sufficient for their validation, contributing to an increase in the overall CVI of the instrument from 0.85 to 0.89 and consequently, the IRR index of the dimension “Resource” from 0.64 to 1.00, as well as the overall IRR of the instrument from 0.94 to 1.00, making the matrix of indicators “QualiAPS Digital—Brazil” validated in all its indicators and dimensions. After validation, a workshop was held with researchers to adjust the wording of the indicators based on the qualitative suggestions of the judges. At that time, it was noted that indicators R5 and III were similar, as were O3 and VI. The most highly rated indicators (III and VI) were maintained and, subsequently, reordered together with the others (check Table 2).

In its final validated version, the matrix “QualiAPS Digital—Brazil” is introduced from the perspective of components, dimensions and indicators. The component “Structure” includes the dimension “Resources” (R), which includes 14 indicators for the quality of care. The component “Processes” includes the dimension “Technical” (T), with four indicators; the dimension “Organizational (O),” with six; and the dimension “Relational” (REL) with three. The component “Results” comprises the dimensions of Short-Term Results (ST), with six indicators, and Medium-Term Results (MT), with four, totaling a matrix with 37 indicators for assessing the quality of digital health care in Brazilian PHC, which can be consulted in the Supplementary material of this article.

**Figure 2:**
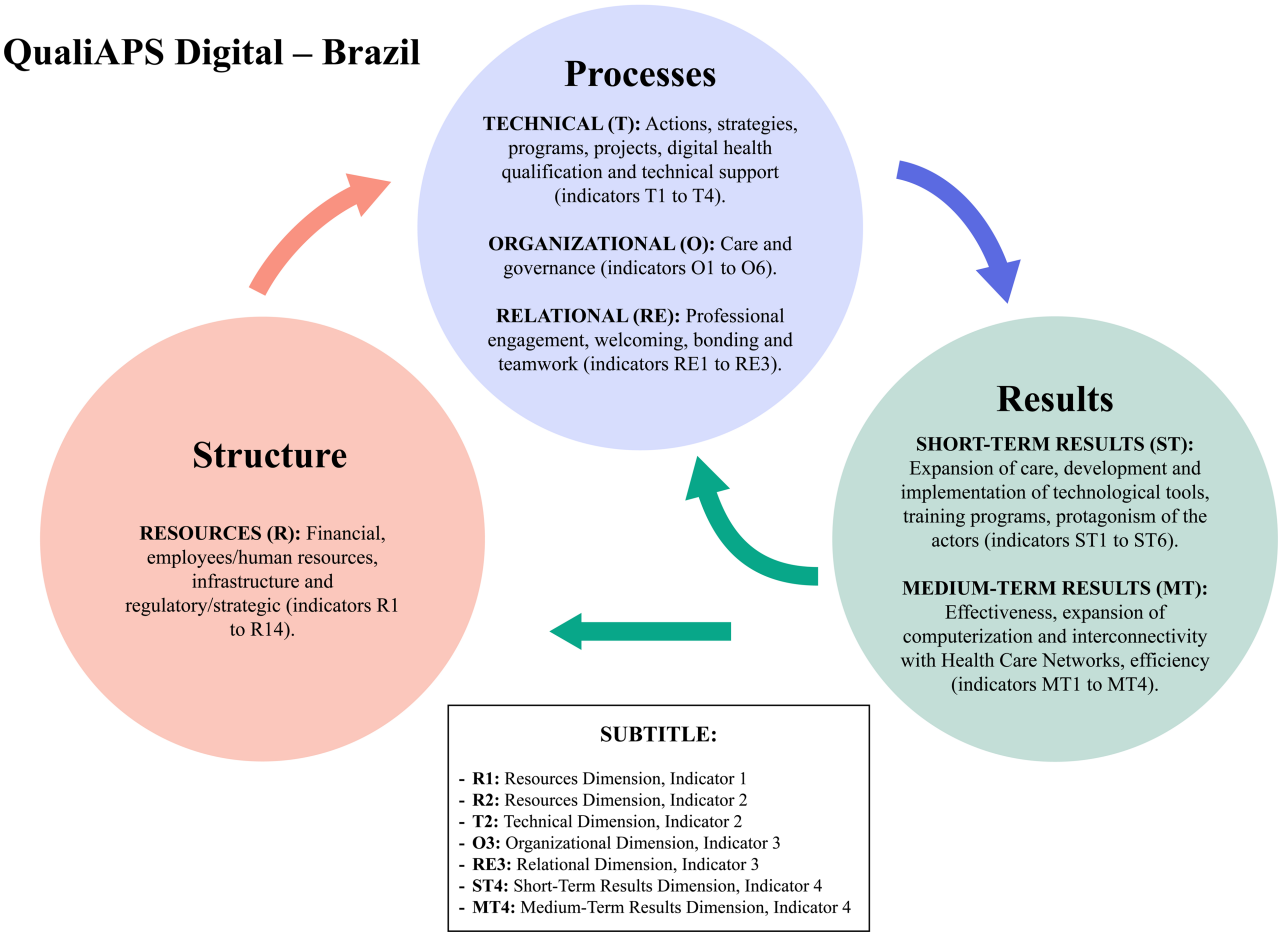
Matrix synthesis and interconnection of components and dimensions.

The “Structure” component, represented by the “Resources” dimension, addresses indicators such as financial aspects, human resources, infrastructure, and regulatory/strategic frameworks outlined in the policy. These indicators support the execution of the “Processes” component, which involves various aspects of care approach such as the “Technical,” “Organizational,” and “Relational” dimensions with their respective indicators, reflecting the way of acting, knowing, supervising, and contributing to the quality of service provision and patient care. Once the “Processes” are executed, the “Results” are generated, explored in the dimensions of “Short-term results” and “Medium-term results,” expressing the effects of the implemented measures. The interconnection among structure, process, and result shows that technological infrastructure, human and financial resources, and policies influence the processes of implementation and use of these technologies, which in turn affect quality, efficiency, expanded access, and patient satisfaction. Positive results feed back into the structure and process, promoting a cycle of continuous improvement. The synthesis of the matrix and the interconnection between the components and dimensions of quality are schematized in Figure 2.

### 4.3 Phase 3: pilot test

The pilot study was attended by one physician, two CHWs, one IT professional and one manager. All of them work in primary health care units, except for the IT professional and the manager, who work in the headquarters of the Municipal Department of Health of the municipality of Campina Grande/Paraíba. Among the participants, three were aged between 30 and 40 years old, one between 50 and 60 and one was aged 60 or older. In terms of education, two had a high school degree and three had a university degree. They were interviewed face-to-face, at their place of work, at a time agreed with each of them. After analyzing the content of the interviews through transcriptions, the authors improved the instruments, refining the pre-existing questions, those that emerged from the interviews, defining the target audience and the respective collection techniques, resulting in five new scripts.

The first, aimed at PHC physicians and nurses, underwent reformulation culminating in the division of this instrument into two different scripts: one questionnaire with 30 objective questions relating to indicators included in the dimensions “Resources,” “Technical” and “Medium-Term Results,” with the suggestion for application in a survey-type study; and 01 interview script containing 32 open questions relating to indicators in the dimensions “Technical,” “Organizational,” “Relational,” “Short-Term Results” and “Medium-Term Results.”

The instrument intended for CHWs was synthesized and resulted in a new script with 30 subjective guiding questions for discussion in the Focus Group, which are related to indicators present in the “Resources,” “Technical,” “Relational” and “Short-Term Results” dimensions. The script aimed at managers remained with the suggestion of an interview, however, there was a reduction and readjustment of the instrument’s content to 28 open questions, alluding to the indicators present in the dimensions “Resource,” “Technical,” “Organizational,” “Short-Term Results” and “Medium-Term Results.” The discussed positions of managers suggested for the interviews were health secretaries, PHC coordinators, health district managers and health unit managers.

The instrument intended for IT professionals remained as an interview script. Nonetheless, questions emerged during the pilot test of this category, which is why it was finalized containing 21 open questions, based on the indicators of the Resources, Technical, Organizational and Medium-Term Results dimensions. The final scripts by technique (survey, interview and focus group) and professional (manager, physician, nurse, CHW and IT professional) covered all the indicators in the matrix of reference assessment proposed in this paper. These documents can be consulted in the Supplementary material of this article.

## 5 Discussion

With the achieved results in this study, a validated instrument is available representing a powerful resource for policy promoters, researchers, managers and health professionals for assessing the quality of care through digital health in Primary Health Care (PHC). This tool is required in terms of assessing processes in the face of the new reality experienced in universal health systems, especially in the Brazilian Unified Health System (SUS, as per its Portuguese acronym), one of the largest universal health care systems in the world.

The instrument “QualiAPS Digital—Brazil” was validated by experts and tested with the target audience, making it possible to produce valid, accurate and interpretable data, thus meeting the requirements of the validation process, as stated by Souza, Alexandre and Guirardello (59). The methodology adopted was the internationally recognized Delphi technique (54), which achieved its goal in two rounds, the minimum required (60).

As recommended by the specialized literature (53), the expert judges in the area of interest of the study analyzed all the proposed indicators, formulated criticisms in relation to their initial version and suggestions for improvement. In the validation process, of the 25 invitations sent electronically in the Delphi first round to the group of the selected experts in digital health assessment, 17 received responses, thus characterizing a return rate of almost 70%, a rate in line with some studies, such as those by Williams and Webb (54), Polit et al. (61), Alexandre and Coluci (62).

Other studies also had a similar sample size and return rate. Brandão et al. (63) listed a total of 13 experts, obtaining feedback from 09, with a response rate of 69%. Batista, Gama and Souza (64) also obtained an approximate response rate in their study (71%), when they sent invitations to 21 and finalized their sample with 15 participants. Still in relation to the number of judges, Martignon et al. (65), Nagarajappa et al. (66), and Shinde et al. (67) used 05, 06 and 15 experts, respectively.

In this study, the abstention rate between rounds was less than 12%, a decrease from 17 to 15 respondents. According to some authors, including Revorêdo et al. (68) and Scarparo et al. (69), the number of participants defined in the initial sample for the Delphi panel must anticipate the abstention rate, which can vary from 20 to 50% between rounds, higher than the values introduced here. It is believed that this low abstention rate can be attributed to the repeated contacts made with the judges, the smaller number of indicators evaluated in the second round, the adequate selection of participants for the sample and the technique used in the study.

In order to make up the sample of experts in this study, criteria such as degree, experience and knowledge on the subject were considered, as well as publications in the area, which are common to other findings by Vieira et al. (70) in an integrative review. Criteria such as skills/knowledge acquired through experience, having specialized skills/knowledge have been consolidated for decades and were already described in the methodological guidelines by Jasper (71).

Following Nair et al. (72) and Vieira et al. (70), regarding the weighting of homogeneity or heterogeneity in the composition of the panel of judges, it was assessed that opting for a heterogeneous group (PHC raters and IT professionals) provided greater variability in the suggested ideas, while taking experience in digital health as a criterion made it possible to make consensus possible without major disagreements around the same item.

In order to determine the reliability of experts when assessing an instrument, it is recommended to use the IRR index in the validation process. The results of this study showed an IRR of 1.00, as recommended by the pertinent literature (56), a fact which, according to Rubio et al. (53), shows a good consensus among the judges in terms of relevance and representativeness. Along the same lines, the CVI index, which was used to assess agreement in relation to the indicators of the instrument, showed satisfactory individual values for the indicators and a final overall result of 0.89, which is also indicated for obtaining validation (56), thus providing more objectivity to the content validity (64).

When addressing the dimensions “Technical,” “Short-Term Results” and “Medium-Term Results,” in their respective indicators, the accomplished validation of the instrument reaffirms Figueiredo et al. (73) in relation to the need for an assessment that contributes to understanding and reflecting on the risks and potential of digital health, considering important aspects of questions such as access, quality of the provided service, reliability and confidentiality of records, as well as public and governmental acceptability of the different employed ICTs.

In the dimensions “Resources,” “Organizational” and “Short-Term Results,” the proposed indicators address crucial aspects for assessing important challenges for the implementation of digital health, such as, for example, infrastructure and coverage; precariousness of health work; convergence with the biomedical model, ethical and political aspects; implementation of advanced technologies; patient acceptance and end-user training in relation to these technologies. These challenges were revealed in various studies (74–76). The health computerization process is occurring worldwide. Accordingly, different evaluation models are being developed (14–21, 77), which shows the concern in evaluating the insertion of digital health in care models.

However, they are not consolidated instruments based on scientifically validated assessment models and do not express the interrelationship between the domains present in each model and their influence on the results. Although they unveil the effects of the use of technologies, they do not consider the impacts on strengthening the health systems where they are implemented. The instrument proposed here considers the context of Brazilian digital health, especially based on Donabedian’s systemic approach, comprising the interconnection of structure (technological infrastructure, human resources, policies), the process of implementing and using technologies and its influence on the results of the use of technologies and impacts on the essential attributes of PHC, as proposed by Starfield (28). It can serve as an evaluation model for other countries, especially low- and middle-income countries that face social determinants of health or contexts similar to those experienced in Brazil.

Taking as a reference the Digital Health Strategy for Brazil (ESD28, as per its Portuguese acronym), which is currently being implemented, it is observed a confluence between the indicators of “QualiAPS Digital—Brazil” and its action plan and priorities: governance and leadership, computerization of the three levels of care, support for improving health care, the user as the protagonist, training and qualification of human resources, interconnectivity environment and innovation ecosystem (14). The proposal of assessment dimensions and indicators is intended to fill the gap in ESD28 in terms of having minimum monitoring and assessment parameters at national level, but which can also include local and regional specificities.

According to Costa and Marin (78), the use of health care technologies must be assessed taking into account aspects of governance, architecture, IT staff, among others, as proposed by the validated matrix, addressing indicators varying from human resources, infrastructure and regulations to short and medium-term results.

In line with the Brazilian context, the instruments cover more than just aspects inherent to the technical and structural concepts of health technologies, since they include sensitive indicators to identify elements of the work process of PHC teams, possible relationships with social and economic vulnerability and digital health usability, as well as human and financial resources.

## 6 Limitations and potentialities of this study

This study had a limitation related to the difficulty in terms of responding to the forms sent out, which prolonged the time intended for data collection, since it adopted virtual and asynchronous data collection, where repeated contacts were made with the judges when the validation forms were not responded to in a certain period. On the other hand, the technique allowed access to geographically distant judges and responses at more opportune times.

Despite the strong theoretical consistency of the instrument, its content was qualitatively validated for use in the context of Primary Health Care in Brazil, a country with continental dimensions and different realities, which limits its degree of reproducibility. However, it can be applied with contextual adaptations, maintaining the addressed analytical dimensions.

The matrix of indicators, validated and tested, has potentialities and the possibility of practical application, thus enabling assessment processes, reflections, changes and/or continuity of professional behaviors, as well as providing a basis for decision-making by managers, with a view to transforming health practices.

## 7 Final considerations

The instrument “QualiAPS Digital—Brazil” consists of a matrix of 37 indicators for the quality of care through digital health, from which guiding themes and/or questions can be extracted to be applied, as was accomplished and suggested in the pilot study described in this article, to the most diverse professionals involved directly or indirectly in PHC care. That said, an instrument that has been validated regarding its relevance, content and theoretical support, as well as tested to assess the quality of care provided through digital health, is now available.

The instrument can be used as a continuous monitoring tool to assess the performance of the employed digital strategies, identify strengths and areas for improvement, as well as assist in strategic processes focused on improving remote primary care provided to the population. In practice, its application will enable actions aimed at the efficiency, effectiveness, safety and humanization of health services, thus seeking excellence in the quality of care.

Further research can be carried out applying this instrument, thus generating scientific data that will enable planning for the implementation of digital health strategies, in line with the elements of the quality of health care and the qualifying attributes for PHC.

## Data availability statement

The original contributions presented in the study are included in the article/Supplementary material, further inquiries can be directed to the corresponding author.

## Ethics statement

The studies involving humans were approved by Research Ethics Committee of the Onofre Lopes University Hospital of the Federal University of Rio Grande do Norte under CAAE no 48655521.9.0000.5292 and protocol number 4.859.682. The studies were conducted in accordance with the local legislation and institutional requirements. The participants provided their written informed consent to participate in this study.

## Author contributions

RCF: Data curation, Formal analysis, Investigation, Methodology, Resources, Supervision, Validation, Visualization, Writing – original draft, Writing – review & editing. ÍSS: Data curation, Formal analysis, Resources, Validation, Visualization, Writing – original draft, Writing – review & editing. AJA: Data curation, Formal analysis, Validation, Visualization, Writing – original draft, Writing – review & editing. CRDVS: Formal analysis, Validation, Visualization, Writing – review & editing. CSM: Data curation, Formal analysis, Supervision, Validation, Visualization, Writing – review & editing. EWGB: Validation, Visualization, Writing – review & editing. PBX: Validation, Visualization, Writing – review & editing. SACU: Conceptualization, Methodology, Project administration, Resources, Supervision, Validation, Visualization, Writing – original draft, Writing – review & editing.
