# Supplementary material for: Preparation and validation of the instrument “QualiAPS digital—Brazil” for assessing digital health care in primary health care: a required tool
Source: Front Public Health. 2024 Jul 16;12:1304148. doi: 10.3389/fpubh.2024.1304148 (PMC11286592; doi:10.3389/fpubh.2024.1304148)
Supplement: Supplementary file 1 [file Data_Sheet_1.PDF]

Database of the 1st Delphi validation round of "QualiAPS digital - Brazil"

JUDGE	Financial resources<br>(R1) Amount and percentage of financial resources transferred to the Municipal Health Secretariat (MHS) by the Ministry of Health (MS) or by other public, private or philanthropic entities to fund digital health actions in Primary Health Care (PHC).	Suggestion/Comment	Collaborators<br>(R2) Number of managers (MHS / PHC / Family Health Unit - FHU) who have implemented a project, program or set of digital health actions per FHU.	Suggestion/Comment
Judge 1	3 - Item needs minor revision to be relevant and appropriate		3 - Item needs minor revision to be relevant and appropriate	
Judge 2	3 - Item needs minor revision to be relevant and appropriate	Percentage of resources allocated to PHC for the costs of digital health actions.	3 - Item needs minor revision to be relevant and appropriate	I think it would be more appropriate to work with percentages (percentage of managers...)
Judge 3	4 - Relevant and appropriate item.		4 - Relevant and appropriate item.	
Judge 4	4 - Relevant and appropriate item.		1 - Item not relevant and not appropriate;	It's not clear what you want to know... I think there are two important pieces of information to look for: the number of programs/projects/actions implemented in the municipality and the number of teams involved in each program/project/action.
Judge 5	3 - Item needs minor revision to be relevant and appropriate	The way it is written, it does not include resources from national programs (e.g. National Leprosy Control Program) or from state actions to be applied to all their municipalities (e.g. computer systems developed and implemented by the State Department of Public Health). If the reference for the application of the tool is the individual analysis of each municipality, the text is adequate. If the reference for application is the state or Brazil, the text would need a little adjustment.	4 - Relevant and appropriate item.	
Judge 6	4 - Relevant and appropriate item.		4 - Relevant and appropriate item.	
Judge 7	4 - Relevant and appropriate item.		4 - Relevant and appropriate item.	
Judge 8	2 Item needs major revision to be relevant and appropriate;	What matters more than the amount is the strategy	4 - Relevant and appropriate item.	
Judge 9	2 Item needs major revision to be relevant and appropriate;		3 - Item needs minor revision to be relevant and appropriate	
Judge 10	4 - Relevant and appropriate item.		4 - Relevant and appropriate item.	
Judge 11	4 - Relevant and appropriate item.		4 - Relevant and appropriate item.	
Judge 12	2 Item needs major revision to be relevant and appropriate;	If it's an open question, it will be difficult for the evaluator to compile the answers and classify the quality. If it's a closed question, I suggest separating it into two fields: one for the quantity and one for the percentage, including the categories for each source of funding.	2 - Item needs major revision to be relevant and appropriate;	If it is an open question, it will be difficult for the evaluator to compile the answers and classify the quality. If it's a closed question, I suggest separating it into three response categories, one for each group of managers (MHS/PHC/FHU).
Judge 13	4 - Relevant and appropriate item.		4 - Relevant and appropriate item.	
Judge 14	4 - Relevant and appropriate item.		3 - Item needs minor revision to be relevant and appropriate	In this item I suggest including whether the manager has a specialization and/or technical qualification in public health and/or health management and/or public health.
Judge 15	4 - Relevant and appropriate item.		4 - Relevant and appropriate item.	
Judge 16	4 - Relevant and appropriate item.		4 - Relevant and appropriate item.	
Judge 17	4 - Relevant and appropriate item.		4 - Relevant and appropriate item.	

Database of the 1st Delphi validation round of "QualiAPS digital - Brazil"

JUDGE	(R3) Number and categories of professionals from the Family Health Teams (e-FHS and e-BH) who develop or have developed digital health actions in the unit/household/community.	Suggestion/Comment	(R4) Number of Information and Communication Technology (ICT) technicians and/or developers involved in digital health actions in Primary Health Care (PHC)	Suggestion/Comment
Judge 1	2 - Item needs major revision to be relevant and appropriate;		2 - Item needs major revision to be relevant and appropriate;	
Judge 2	2 - Item needs major revision to be relevant and appropriate;	I suggest separating this indicator, the number of professionals, and the other, the categories of professional days.	4 - Relevant and appropriate item.	
Judge 3	4 - Relevant and appropriate item.		4 - Relevant and appropriate item.	
Judge 4	4 - Relevant and appropriate item.		4 - Relevant and appropriate item.	
Judge 5	4 - Relevant and appropriate item.		4 - Relevant and appropriate item.	
Judge 6	4 - Relevant and appropriate item.		4 - Relevant and appropriate item.	How do you differentiate between quantity (number of employees) and actual working hours (1 employee 40h/week or 2 employees 20h/week)?
Judge 7	4 - Relevant and appropriate item.		3 - Item needs minor revision to be relevant and appropriate	I suggest changing "involved in the actions" to "offering support to the actions"
Judge 8	4 - Relevant and appropriate item.		3 - Item needs minor revision to be relevant and appropriate	The importance of organization
Judge 9	2 - Item needs major revision to be relevant and appropriate;		2 - Item needs major revision to be relevant and appropriate;	
Judge 10	3 - Item needs minor revision to be relevant and appropriate	We need to know whether this is an isolated and individual action by the professional or a guideline from the municipality's management.	3 - Item needs minor revision to be relevant and appropriate	This is an issue that needs to be looked at in a standardized way. Smaller municipalities cannot be evaluated in the same way as municipalities with greater financial power.
Judge 11	4 - Relevant and appropriate item.		4 - Relevant and appropriate item.	
Judge 12	2 - Item needs major revision to be relevant and appropriate;	If this is an open question, I suggest specifying what these digital health actions are. It may not be clear to the evaluator what the actions are and whether they fall under Digital Health. If it is a closed question, I suggest separating the number and categories of professionals into different questions.	4 - Relevant and appropriate item.	
Judge 13	4 - Relevant and appropriate item.		4 - Relevant and appropriate item.	
Judge 14	4 - Relevant and appropriate item.		3 - Item needs minor revision to be relevant and appropriate	In this item I suggest including whether the technicians and/or developers have a specialization and/or technical qualification in public health and/or health management and/or collective health and/or digital health.
Judge 15	4 - Relevant and appropriate item.		4 - Relevant and appropriate item.	
Judge 16	4 - Relevant and appropriate item.		4 - Relevant and appropriate item.	
Judge 17	4 - Relevant and appropriate item.		3 - Item needs minor revision to be relevant and appropriate	I suggest adjusting the term ICT to DTIC, if appropriate in the context. Digital Information and Communication Technologies (DICT)

Database of the 1st Delphi validation round of "QualiAPS digital - Brazil"

JUDGE	Infrastructure resources (R5) Number of Family Health Units (FHU) that carry out or have carried out digital health actions.	Suggestion/Comment	(R6) Geographical accessibility (possibility of access) and adequacy of the physical spaces of the Family Health Units (FHU) for multiple demands, face-to-face/remote COVID19 and non-COVID19.	Suggestion/Comment
Judge 1	2 - Item needs major revision to be relevant and appropriate;		3 - Item needs minor revision to be relevant and appropriate	
Judge 2	3 - Item needs minor revision to be relevant and appropriate	I'd just leave the verb in the present tense.	2 - Item needs major revision to be relevant and appropriate;	I suggest removing geographical accessibility. "Suitability of spaces ..."
Judge 3	4 - Relevant and appropriate item.		4 - Relevant and appropriate item.	
Judge 4	4 - Relevant and appropriate item.	I suggest the following wording: Number of Family Health units where oral health actions are carried out.	4 - Relevant and appropriate item.	
Judge 5	4 - Relevant and appropriate item.		4 - Relevant and appropriate item.	
Judge 6	4 - Relevant and appropriate item.		4 - Relevant and appropriate item.	
Judge 7	4 - Relevant and appropriate item.		4 - Relevant and appropriate item.	
Judge 8	3 - Item needs minor revision to be relevant and appropriate	it is important to distinguish between sporadic actions and continuity	2 - Item needs major revision to be relevant and appropriate;	Why COVID-19?
Judge 9	2 - Item needs major revision to be relevant and appropriate;		2 - Item needs major revision to be relevant and appropriate;	
Judge 10	3 - Item needs minor revision to be relevant and appropriate	As with R3, it is necessary to check whether this is a coordinated or one-off action, when evaluating	4 - Relevant and appropriate item.	
Judge 11	4 - Relevant and appropriate item.		4 - Relevant and appropriate item.	
Judge 12	3 - Item needs minor revision to be relevant and appropriate	I suggest dividing it into two questions: one about the past and one about the present.	2 - Item needs major revision to be relevant and appropriate;	If it's an open question, it will be difficult for the evaluator to compile the answers and classify the quality. If it is a closed question, I suggest separating it into specific answer categories related to geographical accessibility and what adjustments have been made, including separate answers for face-to-face and remote. I also don't suggest relating it to covid-19.
Judge 13	3 - Item needs minor revision to be relevant and appropriate	This item can be divided into two, specifying the number of FHUs with urban and rural characteristics, since it is known that Digital Health actions take place less frequently in rural areas due to connectivity problems, among others.	4 - Relevant and appropriate item.	
Judge 14	3 - Item needs minor revision to be relevant and appropriate	Important to include. What digital health actions are developed.	3 - Item needs minor revision to be relevant and appropriate	
Judge 15	4 - Relevant and appropriate item.	I suggest that, in addition to the number of actions, they should also be listed.	4 - Relevant and appropriate item.	
Judge 16	4 - Relevant and appropriate item.		3 - Item needs minor revision to be relevant and appropriate	As the instrument refers to ICT, it would be interesting to pay attention to remote accessibility, in terms of the internet or other means of virtual access
Judge 17	4 - Relevant and appropriate item.		4 - Relevant and appropriate item.	

Database of the 1st Delphi validation round of "QualiAPS digital - Brazil"

JUDGE	(R7) Access to and quality of equipment and tools for operationalizing digital health (computers, notebooks, tablets, wireless routers, external hard drives, and network servers in the UBS.	Suggestion/Comment	(R8) Availability and quality of the internet, connectivity (computers with access to the internet network), and integration between systems (internet of things such as sensors, intelligent monitoring that can be viewed on several devices at the same time).	Suggestion/Comment
Judge 1	2 - Item needs major revision to be relevant and appropriate;		2 - Item needs major revision to be relevant and appropriate;	
Judge 2	4 - Relevant and appropriate item.		2 - Item needs major revision to be relevant and appropriate;	Integration between systems should be a separate indicator.
Judge 3	4 - Relevant and appropriate item.		4 - Relevant and appropriate item.	
Judge 4	4 - Relevant and appropriate item.		4 - Relevant and appropriate item.	
Judge 5	4 - Relevant and appropriate item.		4 - Relevant and appropriate item.	
Judge 6	4 - Relevant and appropriate item.		4 - Relevant and appropriate item.	
Judge 7	3 - Item needs minor revision to be relevant and appropriate	I believe that network servers are included in R8	4 - Relevant and appropriate item.	
Judge 8	3 - Item needs minor revision to be relevant and appropriate		3 - Item needs minor revision to be relevant and appropriate	
Judge 9	2 - Item needs major revision to be relevant and appropriate;		2 - Item needs major revision to be relevant and appropriate;	
Judge 10	4 - Relevant and appropriate item.	Internet quality should be associated with this issue, which is the basis for access to health services.	2 - Item needs major revision to be relevant and appropriate;	It's not clear what is meant by "integration". Which systems should be integrated and how?
Judge 11	4 - Relevant and appropriate item.	I suggest reading the thesis by Rodrigo Cândido Borges from UFG, supervised by Maria Márcia Bachion. The thesis includes other variables that may help you.	4 - Relevant and appropriate item.	
Judge 12	2 - Item needs major revision to be relevant and appropriate;	If it's an open question, it will be difficult for the evaluator to compile the answers and classify the quality. If it's a closed question, I suggest separating it into categories for each type of equipment.	2 - Item needs major revision to be relevant and appropriate;	If it's an open question, it will be difficult for the evaluator to compile the answers and classify the quality. If it's a closed question, I suggest separating it into categories for each item mentioned in the statement.
Judge 13	4 - Relevant and appropriate item.	Institutional equipment and tools, not personal ones.	4 - Relevant and appropriate item.	
Judge 14	4 - Relevant and appropriate item.		4 - Relevant and appropriate item.	
Judge 15	4 - Relevant and appropriate item.		4 - Relevant and appropriate item.	
Judge 16	4 - Relevant and appropriate item.		4 - Relevant and appropriate item.	
Judge 17	4 - Relevant and appropriate item.		4 - Relevant and appropriate item.	

Database of the 1st Delphi validation round of "QualiAPS digital - Brazil"

JUDGE	(R9) Digital tools (ICT) used: phone calls, videos, text messages via apps, social media, portals, cloud computing ("cloud" data processing on the internet)	Suggestion/Comment	(R10) Quality of information systems, data management and interface with technology users (health professionals and users)	Suggestion/Comment
Judge 1	2 - Item needs major revision to be relevant and appropriate;		2 - Item needs major revision to be relevant and appropriate;	
Judge 2	4 - Relevant and appropriate item.		4 - Relevant and appropriate item.	
Judge 3	4 - Relevant and appropriate item.		4 - Relevant and appropriate item.	
Judge 4	4 - Relevant and appropriate item.		1 - Item not relevant and not appropriate;	
Judge 5	4 - Relevant and appropriate item.		4 - Relevant and appropriate item.	
Judge 6	4 - Relevant and appropriate item.		4 - Relevant and appropriate item.	More abstract concepts such as quality and management can have different interpretations for each person.
Judge 7	3 - Item needs minor revision to be relevant and appropriate	Add e-mail and social networks.	4 - Relevant and appropriate item.	
Judge 8	3 - Item needs minor revision to be relevant and appropriate		2 - Item needs major revision to be relevant and appropriate;	How do you define quality?
Judge 9	2 - Item needs major revision to be relevant and appropriate;		2 - Item needs major revision to be relevant and appropriate;	
Judge 10	4 - Relevant and appropriate item.		4 - Relevant and appropriate item.	
Judge 11	4 - Relevant and appropriate item.		4 - Relevant and appropriate item.	
Judge 12	2 - Item needs major revision to be relevant and appropriate;	If it's an open question, it will be difficult for the evaluator to compile the answers and classify the quality. If it's a closed question, I suggest separating it into categories for each item mentioned in the statement.	2 - Item needs major revision to be relevant and appropriate;	If it's an open question, it will be difficult for the evaluator to compile the answers and classify the quality. If it's a closed question, I suggest separating it into categories for each item mentioned in the statement.
Judge 13	4 - Relevant and appropriate item.		4 - Relevant and appropriate item.	
Judge 14	4 - Relevant and appropriate item.		4 - Relevant and appropriate item.	
Judge 15	4 - Relevant and appropriate item.		3 - Item needs minor revision to be relevant and appropriate	The question is confusing.
Judge 16	4 - Relevant and appropriate item.		4 - Relevant and appropriate item.	
Judge 17	3 - Item needs minor revision to be relevant and appropriate	I suggest adjusting the term ICT to TDIC, if appropriate in the context.	4 - Relevant and appropriate item.	

Database of the 1st Delphi validation round of "QualiAPS digital - Brazil"

JUDGE	(R11) Existence of technical assistance for digital services provided by Family Health Units.	Suggestion/Comment	Regulatory/strategic resources (R12) Partnerships (state/municipal telehealth centers; universities; other ICT centers.	Suggestion/Comment
Judge 1	2 - Item needs major revision to be relevant and appropriate;		2 - Item needs major revision to be relevant and appropriate;	
Judge 2	3 - Item needs minor revision to be relevant and appropriate	Was R04 unable to meet this indicator? If so, I suggest keeping R04.	3 - Item needs minor revision to be relevant and appropriate	Improve the wording of the indicator
Judge 3	4 - Relevant and appropriate item.		4 - Relevant and appropriate item.	
Judge 4	4 - Relevant and appropriate item.		4 - Relevant and appropriate item.	
Judge 5	4 - Relevant and appropriate item.		4 - Relevant and appropriate item.	
Judge 6	4 - Relevant and appropriate item.		4 - Relevant and appropriate item.	
Judge 7	3 - Item needs minor revision to be relevant and appropriate	Adapt to "Existence of a technical assistance service for digital health actions by the FHS"	3 - Item needs minor revision to be relevant and appropriate	Put Telehealth and Telemedicine.
Judge 8	3 - Item needs minor revision to be relevant and appropriate		4 - Relevant and appropriate item.	
Judge 9	2 - Item needs major revision to be relevant and appropriate;		2 - Item needs major revision to be relevant and appropriate;	
Judge 10	4 - Relevant and appropriate item.		4 - Relevant and appropriate item.	In this case, you want to know if you have a partnership or not, right?
Judge 11	4 - Relevant and appropriate item.		4 - Relevant and appropriate item.	
Judge 12	4 - Relevant and appropriate item.		2 - Item needs major revision to be relevant and appropriate;	If it's an open question, it will be difficult for the evaluator to compile the answers and classify the quality. If it's a closed question, I suggest separating it into categories for each item mentioned in the statement.
Judge 13	4 - Relevant and appropriate item.		4 - Relevant and appropriate item.	
Judge 14	4 - Relevant and appropriate item.		4 - Relevant and appropriate item.	
Judge 15	3 - Item needs minor revision to be relevant and appropriate	Technical assistance for what? Sofware? Hardware? Networking?	4 - Relevant and appropriate item.	
Judge 16	4 - Relevant and appropriate item.		4 - Relevant and appropriate item.	
Judge 17	4 - Relevant and appropriate item.		3 - Item needs minor revision to be relevant and appropriate	I suggest adjusting the term ICT to TDIC, if appropriate in the context.

Database of the 1st Delphi validation round of "QualiAPS digital - Brazil"

JUDGE	(R13) Information system including digital health actions	Suggestion/Comment	(R14) Existence and adequacy of protocols, guidelines and regulations for the organization of digital health actions.	Suggestion/Comment
Judge 1	2 - Item needs major revision to be relevant and appropriate;		3 - Item needs minor revision to be relevant and appropriate	
Judge 2	4 - Relevant and appropriate item.		4 - Relevant and appropriate item.	
Judge 3	4 - Relevant and appropriate item.		4 - Relevant and appropriate item.	
Judge 4	4 - Relevant and appropriate item.		4 - Relevant and appropriate item.	
Judge 5	4 - Relevant and appropriate item.		4 - Relevant and appropriate item.	
Judge 6	4 - Relevant and appropriate item.		4 - Relevant and appropriate item.	
Judge 7	4 - Relevant and appropriate item.		4 - Relevant and appropriate item.	
Judge 8	3 - Item needs minor revision to be relevant and appropriate		4 - Relevant and appropriate item.	
Judge 9	2 - Item needs major revision to be relevant and appropriate;		2 - Item needs major revision to be relevant and appropriate;	
Judge 10	2 - Item needs major revision to be relevant and appropriate;	What would be "inclusion of digital health actions"?	4 - Relevant and appropriate item.	
Judge 11	2 - Item needs major revision to be relevant and appropriate;	I don't understand. Is it related to social inclusion, or to the PCD?	4 - Relevant and appropriate item.	I suggest adding aspects related to the physical environment conducive to equipment maintenance (useful life) to the structure.
Judge 12	2 - Item needs major revision to be relevant and appropriate;	If the "inclusion of digital health actions" is about interoperability, should the question be more direct?	2 - Item needs major revision to be relevant and appropriate;	If it's an open question, it will be difficult for the evaluator to compile the answers and rate the quality. If it's a closed question, I suggest separating it into two questions: whether it exists and whether it was adequate.
Judge 13	4 - Relevant and appropriate item.		4 - Relevant and appropriate item.	
Judge 14	4 - Relevant and appropriate item.		4 - Relevant and appropriate item.	
Judge 15	4 - Relevant and appropriate item.		4 - Relevant and appropriate item.	
Judge 16	4 - Relevant and appropriate item.		4 - Relevant and appropriate item.	
Judge 17	4 - Relevant and appropriate item.		4 - Relevant and appropriate item.	

Database of the 1st Delphi validation round of "QualiAPS digital - Brazil"

JUDGE	(T1) Provision of individual and collective digital health actions carried out by professionals from Family Health Teams (e-SF or e-AB) for health maintenance, from promotion, protection, prevention, diagnosis, to treatment, rehabilitation, palliative care and harm reduction, indications (COVID 19 and/or other clinical or epidemiological conditions) categories of professionals involved, target population and periodicity (before and/or after the start of COVID 19, only during COVID 19 social distancing measures, or with continuity with the suspension of these measures.	Suggestion/Comment	(T2) Strategies, programs and projects developed inside and outside the physical spaces of health units	Suggestion/Comment
Judge 1	4 - Relevant and appropriate item.		3 - Item needs minor revision to be relevant and appropriate	
Judge 2	2 - Item needs major revision to be relevant and appropriate;	Very confusing indicator. I suggest splitting it up.	3 - Item needs minor revision to be relevant and appropriate	Strategies, programs and projects related to digital health? If yes. You could improve the wording.
Judge 3	4 - Relevant and appropriate item.		4 - Relevant and appropriate item.	
Judge 4	3 - Item needs minor revision to be relevant and appropriate	I suggest replacing the term "for health maintenance" with "for health care". Wouldn't it be a case of dismembering this indicator?	3 - Item needs minor revision to be relevant and appropriate	add "in digital health" after projects...
Judge 5	4 - Relevant and appropriate item.		4 - Relevant and appropriate item.	
Judge 6	4 - Relevant and appropriate item.		3 - Item needs minor revision to be relevant and appropriate	What is the focus/area of these strategies, programs and projects? I suggest specifying to avoid confusion.
Judge 7	3 - Item needs minor revision to be relevant and appropriate	It's very dense. The category of professionals, population and periodicity could be another indicator.	4 - Relevant and appropriate item.	
Judge 8	2 - Item needs major revision to be relevant and appropriate;	confused	3 - Item needs minor revision to be relevant and appropriate	strategies are different from projects
Judge 9	3 - Item needs minor revision to be relevant and appropriate		3 - Item needs minor revision to be relevant and appropriate	
Judge 10	4 - Relevant and appropriate item.		4 - Relevant and appropriate item.	
Judge 11	3 - Item needs minor revision to be relevant and appropriate	The protocols in the previous question are also part of processes. I suggest separating the items by action: one specifically for promotion, another exclusively for prevention, etc.	4 - Relevant and appropriate item.	
Judge 12	2 - Item needs major revision to be relevant and appropriate;	If it's an open question, it will be difficult for the evaluator to compile the answers and classify the quality. If it's a closed question, I suggest separating it into categories for each item mentioned in the statement.	2 - Item needs major revision to be relevant and appropriate;	If it's an open question, it will be difficult for the evaluator to compile the answers and classify the quality. If it's a closed question, I suggest separating it into categories for each item mentioned in the statement.
Judge 13	4 - Relevant and appropriate item.		4 - Relevant and appropriate item.	
Judge 14	4 - Relevant and appropriate item.		4 - Relevant and appropriate item.	
Judge 15	4 - Relevant and appropriate item.		4 - Relevant and appropriate item.	
Judge 16	4 - Relevant and appropriate item.	Interesting that you put a after () the word indications	4 - Relevant and appropriate item.	
Judge 17	4 - Relevant and appropriate item.		4 - Relevant and appropriate item.	

Database of the 1st Delphi validation round of "QualiAPS digital - Brazil"

JUDGE	(T3) Provision and qualification of health professionals and managers for continuing health education using technological resources and aiming to expand their use among professionals and users.	Suggestion/Comment	(T4) Adequate technical support for the use of technologies with guaranteed security and protection of personal data	Suggestion/Comment
Judge 1	3 - Item needs minor revision to be relevant and appropriate		2 - Item needs major revision to be relevant and appropriate;	
Judge 2	4 - Relevant and appropriate item.		4 - Relevant and appropriate item.	
Judge 3	4 - Relevant and appropriate item.		4 - Relevant and appropriate item.	
Judge 4	4 - Relevant and appropriate item.		3 - Item needs minor revision to be relevant and appropriate	
Judge 5	4 - Relevant and appropriate item.		4 - Relevant and appropriate item.	
Judge 6	4 - Relevant and appropriate item.		4 - Relevant and appropriate item.	
Judge 7	3 - Item needs minor revision to be relevant and appropriate	I suggest removing "offer" and leaving the rest as it is.	4 - Relevant and appropriate item.	
Judge 8	3 - Item needs minor revision to be relevant and appropriate		3 - Item needs minor revision to be relevant and appropriate	
Judge 9	2 - Item needs major revision to be relevant and appropriate;		2 - Item needs major revision to be relevant and appropriate;	
Judge 10	4 - Relevant and appropriate item.		4 - Relevant and appropriate item.	
Judge 11	3 - Item needs minor revision to be relevant and appropriate	Make an item with permanent education.	2 - Item needs major revision to be relevant and appropriate;	When talking about security, extrapolate to access to server rooms and reception. Entrance to the building. Physical security. With regard to this item, I would have to specify what technical support would be specifically, as we have managers who are not from the Health or ICT areas.
Judge 12	2 - Item needs major revision to be relevant and appropriate;	If it's an open question, it will be difficult for the evaluator to compile the answers and classify the quality. If it's a closed question, I suggest separating it into specific questions: first if there are any, then which ones.	4 - Relevant and appropriate item.	
Judge 13	4 - Relevant and appropriate item.		4 - Relevant and appropriate item.	
Judge 14	4 - Relevant and appropriate item.		4 - Relevant and appropriate item.	
Judge 15	4 - Relevant and appropriate item.		4 - Relevant and appropriate item.	
Judge 16	4 - Relevant and appropriate item.		4 - Relevant and appropriate item.	
Judge 17	3 - Item needs minor revision to be relevant and appropriate	I suggest using the term 'permanent health education' to replace the term 'continuing education'.	4 - Relevant and appropriate item.	

Database of the 1st Delphi validation round of "QualiAPS digital - Brazil"

JUDGE	Care<br>(O1) Provision of digital health actions aligned with guarantees of access, comprehensive and longitudinal care, coordination and ordering of care, family and community orientation and cultural competence	Suggestion/Comment	(O2) Health surveillance actions through ICT in the territories to support vulnerable groups	Suggestion/Comment
Judge 1	3 - Item needs minor revision to be relevant and appropriate		2 - Item needs major revision to be relevant and appropriate;	
Judge 2	4 - Relevant and appropriate item.		4 - Relevant and appropriate item.	
Judge 3	4 - Relevant and appropriate item.		4 - Relevant and appropriate item.	
Judge 4	4 - Relevant and appropriate item.		4 - Relevant and appropriate item.	
Judge 5	4 - Relevant and appropriate item.		4 - Relevant and appropriate item.	
Judge 6	4 - Relevant and appropriate item.		4 - Relevant and appropriate item.	
Judge 7	4 - Relevant and appropriate item.		4 - Relevant and appropriate item.	
Judge 8	3 - Item needs minor revision to be relevant and appropriate		4 - Relevant and appropriate item.	
Judge 9	2 - Item needs major revision to be relevant and appropriate;		2 - Item needs major revision to be relevant and appropriate;	
Judge 10	4 - Relevant and appropriate item.		4 - Relevant and appropriate item.	
Judge 11	3 - Item needs minor revision to be relevant and appropriate	To make it easier to organize your database, this item would need to be categorized.	4 - Relevant and appropriate item.	
Judge 12	2 - Item needs major revision to be relevant and appropriate;	If it's an open question, it will be difficult for the evaluator to compile the answers and classify the quality. If it's a closed question, I suggest separating it into categories for each item mentioned in the statement.	4 - Relevant and appropriate item.	
Judge 13	4 - Relevant and appropriate item.		4 - Relevant and appropriate item.	
Judge 14	4 - Relevant and appropriate item.		4 - Relevant and appropriate item.	
Judge 15	4 - Relevant and appropriate item.		4 - Relevant and appropriate item.	
Judge 16	4 - Relevant and appropriate item.		4 - Relevant and appropriate item.	
Judge 17	4 - Relevant and appropriate item.		3 - Item needs minor revision to be relevant and appropriate	I suggest adjusting the term ICT to TDIC, if appropriate in the context.

Database of the 1st Delphi validation round of "QualiAPS digital - Brazil"

JUDGE	Governance<br>(O3) Interoperability conditions for operational systems, such as interoperability between the Primary Care Health Information System - SISAB and its applications (Electronic Citizen Record - PEC, e-SUS Territory and Collective Activity), and between other systems and applications used by care professionals.	Suggestion/Comment	(O4) Planning the feasibility of technological solutions for health demands in PHC with monitoring and management of resources.	Suggestion/Comment
Judge 1	2 - Item needs major revision to be relevant and appropriate;		2 - Item needs major revision to be relevant and appropriate;	
Judge 2	4 - Relevant and appropriate item.		4 - Relevant and appropriate item.	
Judge 3	4 - Relevant and appropriate item.		4 - Relevant and appropriate item.	
Judge 4	4 - Relevant and appropriate item.		4 - Relevant and appropriate item.	
Judge 5	4 - Relevant and appropriate item.		4 - Relevant and appropriate item.	
Judge 6	4 - Relevant and appropriate item.		4 - Relevant and appropriate item.	Perhaps this item could be expanded into one or two more items with specific indications that are encompassed by the terms "planning" and "feasibility"?
Judge 7	4 - Relevant and appropriate item.		4 - Relevant and appropriate item.	
Judge 8	3 - Item needs minor revision to be relevant and appropriate	confusion of concepts	3 - Item needs minor revision to be relevant and appropriate	Feasibility planning: what does this mean?
Judge 9	2 - Item needs major revision to be relevant and appropriate;		2 - Item needs major revision to be relevant and appropriate;	
Judge 10	4 - Relevant and appropriate item.		4 - Relevant and appropriate item.	
Judge 11	4 - Relevant and appropriate item.		4 - Relevant and appropriate item.	
Judge 12	4 - Relevant and appropriate item.		4 - Relevant and appropriate item.	
Judge 13	4 - Relevant and appropriate item.		3 - Item needs minor revision to be relevant and appropriate	It needs a better description, with a practical example.
Judge 14	4 - Relevant and appropriate item.		4 - Relevant and appropriate item.	
Judge 15	4 - Relevant and appropriate item.		4 - Relevant and appropriate item.	
Judge 16	4 - Relevant and appropriate item.		4 - Relevant and appropriate item.	
Judge 17	4 - Relevant and appropriate item.		4 - Relevant and appropriate item.	

Database of the 1st Delphi validation round of "QualiAPS digital - Brazil"

JUDGE	(O5) Actions to encourage innovations in management, care and educational technologies on the part of the Municipal Health Secretariat (MHS).	Suggestion/Comment	(O6) MHS partnerships for research into digital solutions to health problems in PHC.	Suggestion/Comment	(RE1) Professional engagement and community participation in the choice, implementation and evaluation of technologies.	Suggestion/Comment
Judge 1	2 - Item needs major revision to be relevant and appropriate;		2 - Item needs major revision to be relevant and appropriate;		2 - Item needs major revision to be relevant and appropriate;	
Judge 2	4 - Relevant and appropriate item.		2 - Item needs major revision to be relevant and appropriate;	Replace search with actions	4 - Relevant and appropriate item.	
Judge 3	4 - Relevant and appropriate item.		4 - Relevant and appropriate item.		4 - Relevant and appropriate item.	
Judge 4	4 - Relevant and appropriate item.		4 - Relevant and appropriate item.		4 - Relevant and appropriate item.	
Judge 5	4 - Relevant and appropriate item.		4 - Relevant and appropriate item.		4 - Relevant and appropriate item.	
Judge 6	4 - Relevant and appropriate item.		4 - Relevant and appropriate item.		4 - Relevant and appropriate item.	
Judge 7	3 - Item needs minor revision to be relevant and appropriate	What kind of incentives: financial? Improvement?	4 - Relevant and appropriate item.		4 - Relevant and appropriate item.	
Judge 8	3 - Item needs minor revision to be relevant and appropriate	Why only the Municipal Health Secretariat?	3 - Item needs minor revision to be relevant and appropriate	before solutions we need to know the problems	3 - Item needs minor revision to be relevant and appropriate	confused: mixing two different things
Judge 9	2 - Item needs major revision to be relevant and appropriate;		2 - Item needs major revision to be relevant and appropriate;		3 - Item needs minor revision to be relevant and appropriate	
Judge 10	4 - Relevant and appropriate item.		4 - Relevant and appropriate item.		4 - Relevant and appropriate item.	
Judge 11	4 - Relevant and appropriate item.		4 - Relevant and appropriate item.		4 - Relevant and appropriate item.	
Judge 12	4 - Relevant and appropriate item.		4 - Relevant and appropriate item.		4 - Relevant and appropriate item.	
Judge 13	4 - Relevant and appropriate item.		4 - Relevant and appropriate item.		4 - Relevant and appropriate item.	
Judge 14	4 - Relevant and appropriate item.		4 - Relevant and appropriate item.		4 - Relevant and appropriate item.	
Judge 15	4 - Relevant and appropriate item.		4 - Relevant and appropriate item.		4 - Relevant and appropriate item.	
Judge 16	4 - Relevant and appropriate item.		4 - Relevant and appropriate item.		4 - Relevant and appropriate item.	
Judge 17	4 - Relevant and appropriate item.		4 - Relevant and appropriate item.		4 - Relevant and appropriate item.	

Database of the 1st Delphi validation round of "QualiAPS digital - Brazil"

JUDGE	(RE2) Reinforcement of reception and bonding with users, even with the technology interface	Suggestion/Comment	(RE3) Creativity and willingness of managers and professionals to improve teamwork.	Suggestion/Comment
Judge 1	2 - Item needs major revision to be relevant and appropriate;		2 - Item needs major revision to be relevant and appropriate;	
Judge 2	4 - Relevant and appropriate item.		4 - Relevant and appropriate item.	
Judge 3	4 - Relevant and appropriate item.		4 - Relevant and appropriate item.	
Judge 4	3 - Item needs minor revision to be relevant and appropriate	I suggest removing the term "even with"... replacing it with: through the interface of technology	2 - Item needs major revision to be relevant and appropriate;	unspecific... would it be "use of a technology interface with an impact on improving teamwork? Is that what you're looking for?
Judge 5	4 - Relevant and appropriate item.		4 - Relevant and appropriate item.	
Judge 6	4 - Relevant and appropriate item.		4 - Relevant and appropriate item.	
Judge 7	4 - Relevant and appropriate item.		4 - Relevant and appropriate item.	
Judge 8	3 - Item needs minor revision to be relevant and appropriate	Foster care: how do you measure it?	3 - Item needs minor revision to be relevant and appropriate	how is it measured?
Judge 9	3 - Item needs minor revision to be relevant and appropriate		3 - Item needs minor revision to be relevant and appropriate	
Judge 10	4 - Relevant and appropriate item.		4 - Relevant and appropriate item.	
Judge 11	4 - Relevant and appropriate item.		4 - Relevant and appropriate item.	
Judge 12	4 - Relevant and appropriate item.		2 - Item needs major revision to be relevant and appropriate;	What would it be like to assess the creativity and willingness of a manager? What would the criteria be?
Judge 13	4 - Relevant and appropriate item.		3 - Item needs minor revision to be relevant and appropriate	In addition to willingness (proactivity), the intentionality of PHC workers can be listed.
Judge 14	4 - Relevant and appropriate item.		4 - Relevant and appropriate item.	
Judge 15	4 - Relevant and appropriate item.		4 - Relevant and appropriate item.	
Judge 16	4 - Relevant and appropriate item.		4 - Relevant and appropriate item.	
Judge 17	4 - Relevant and appropriate item.		4 - Relevant and appropriate item.	

Database of the 1st Delphi validation round of "QualiAPS digital - Brazil"

JUDGE	(CP1) Expanding the supply of care by choosing the most appropriate practices, technologies and instruments to support the lines of care, based on success stories.	Suggestion/Comment	(CP2) More accessible and sustainable services	Suggestion/Comment
Judge 1	2 - Item needs major revision to be relevant and appropriate;		2 - Item needs major revision to be relevant and appropriate;	
Judge 2	4 - Relevant and appropriate item.		3 - Item needs minor revision to be relevant and appropriate	With the help of ICT? Which services?
Judge 3	4 - Relevant and appropriate item.		4 - Relevant and appropriate item.	
Judge 4	4 - Relevant and appropriate item.		4 - Relevant and appropriate item.	
Judge 5	4 - Relevant and appropriate item.		4 - Relevant and appropriate item.	
Judge 6	4 - Relevant and appropriate item.		4 - Relevant and appropriate item.	However, I would suggest better qualifying "Services"
Judge 7	4 - Relevant and appropriate item.		1 - Item not relevant and not appropriate;	I believe that other existing indicators will already reveal this.
Judge 8	4 - Relevant and appropriate item.		3 - Item needs minor revision to be relevant and appropriate	how to measure?
Judge 9	3 - Item needs minor revision to be relevant and appropriate		3 - Item needs minor revision to be relevant and appropriate	
Judge 10	4 - Relevant and appropriate item.		3 - Item needs minor revision to be relevant and appropriate	It is necessary to clarify the responsibility and the type of accessibility and sustainability you want to measure.
Judge 11	4 - Relevant and appropriate item.		4 - Relevant and appropriate item.	
Judge 12	2 - Item needs major revision to be relevant and appropriate;	If it's an open question, it will be difficult for the evaluator to compile the answers and classify quality. If it's a closed question, I suggest separating it into two categories: one for information on whether there has been an expansion in the provision of care, and another to assess whether the practices, technologies and instruments are adequate.	2 - Item needs major revision to be relevant and appropriate;	How do you identify a service as more accessible and sustainable?
Judge 13	4 - Relevant and appropriate item.		3 - Item needs minor revision to be relevant and appropriate	I suggest a better description of this item, citing an example of this type of service.
Judge 14	4 - Relevant and appropriate item.		4 - Relevant and appropriate item.	
Judge 15	4 - Relevant and appropriate item.		4 - Relevant and appropriate item.	
Judge 16	4 - Relevant and appropriate item.		4 - Relevant and appropriate item.	
Judge 17	4 - Relevant and appropriate item.		4 - Relevant and appropriate item.	

Database of the 1st Delphi validation round of "QualiAPS digital - Brazil"

JUDGE	(CP3) Development and implementation of synchronous, asynchronous and monitoring technological tools, fostering management, care and educational actions from a PHC perspective.	Suggestion/Comment	(CP4) Institutionalization of training and continuing education programs including digital health for PHC professionals and users.	Suggestion/Comment
Judge 1	2 - Item needs major revision to be relevant and appropriate;		2 - Item needs major revision to be relevant and appropriate;	
Judge 2	4 - Relevant and appropriate item.		4 - Relevant and appropriate item.	
Judge 3	4 - Relevant and appropriate item.		4 - Relevant and appropriate item.	
Judge 4	4 - Relevant and appropriate item.		4 - Relevant and appropriate item.	
Judge 5	4 - Relevant and appropriate item.		4 - Relevant and appropriate item.	
Judge 6	4 - Relevant and appropriate item.		4 - Relevant and appropriate item.	
Judge 7	4 - Relevant and appropriate item.		4 - Relevant and appropriate item.	
Judge 8	2 - Item needs major revision to be relevant and appropriate;	confused with the first indicator	4 - Relevant and appropriate item.	
Judge 9	2 - Item needs major revision to be relevant and appropriate;		2 - Item needs major revision to be relevant and appropriate;	
Judge 10	4 - Relevant and appropriate item.		4 - Relevant and appropriate item.	
Judge 11	4 - Relevant and appropriate item.		3 - Item needs minor revision to be relevant and appropriate	Continuing education too.
Judge 12	4 - Relevant and appropriate item.		4 - Relevant and appropriate item.	
Judge 13	4 - Relevant and appropriate item.		4 - Relevant and appropriate item.	
Judge 14	4 - Relevant and appropriate item.		4 - Relevant and appropriate item.	
Judge 15	4 - Relevant and appropriate item.		4 - Relevant and appropriate item.	
Judge 16	4 - Relevant and appropriate item.		4 - Relevant and appropriate item.	
Judge 17	4 - Relevant and appropriate item.		3 - Item needs minor revision to be relevant and appropriate	I suggest using the term permanent education.

Database of the 1st Delphi validation round of "QualiAPS digital - Brazil"

JUDGE	(CP5) Active governance system connected to the needs of the services, incentives to expand digital health programs, quality improvement cycles and evaluative research.	Suggestion/Comment	(CP6) Protagonism of the actors involved: clear definition of responsibilities, trust in the technologies, satisfaction of the actors involved and practices within regulatory and ethical standards.	Suggestion/Comment
Judge 1	2 - Item needs major revision to be relevant and appropriate;		2 - Item needs major revision to be relevant and appropriate;	
Judge 2	4 - Relevant and appropriate item.		4 - Relevant and appropriate item.	
Judge 3	4 - Relevant and appropriate item.		4 - Relevant and appropriate item.	
Judge 4	4 - Relevant and appropriate item.		4 - Relevant and appropriate item.	
Judge 5	4 - Relevant and appropriate item.		4 - Relevant and appropriate item.	
Judge 6	4 - Relevant and appropriate item.		4 - Relevant and appropriate item.	
Judge 7	4 - Relevant and appropriate item.		4 - Relevant and appropriate item.	
Judge 8	3 - Item needs minor revision to be relevant and appropriate	mixing different concepts	3 - Item needs minor revision to be relevant and appropriate	what is trust in technology?
Judge 9	2 - Item needs major revision to be relevant and appropriate;		2 - Item needs major revision to be relevant and appropriate;	
Judge 10	4 - Relevant and appropriate item.		4 - Relevant and appropriate item.	
Judge 11	4 - Relevant and appropriate item.		4 - Relevant and appropriate item.	
Judge 12	2 - Item needs major revision to be relevant and appropriate;	The question is very broad. How do you classify a system of active and connected governance?	4 - Relevant and appropriate item.	
Judge 13	4 - Relevant and appropriate item.		4 - Relevant and appropriate item.	
Judge 14	4 - Relevant and appropriate item.		4 - Relevant and appropriate item.	
Judge 15	4 - Relevant and appropriate item.		4 - Relevant and appropriate item.	
Judge 16	4 - Relevant and appropriate item.		4 - Relevant and appropriate item.	
Judge 17	4 - Relevant and appropriate item.		4 - Relevant and appropriate item.	

Database of the 1st Delphi validation round of "QualiAPS digital - Brazil"

JUDGE	(MP1) Effectiveness (capacity to produce improvement) of digital health in the quality of care offered, with positive effects on the adherence of the actors involved, satisfaction/acceptability of health users, resolutiveness, technical accuracy (pertinence of the choices of technologies in relation to health problems), continuity and coordination of care in the Health Care Networks (RAS).	Suggestion/Comment	(MP2) Expansion of computerization in PHC: strengthening information systems with data integrity (reliability and consistency of information throughout its useful life cycle) and operating systems.	Suggestion/Comment
Judge 1	2 - Item needs major revision to be relevant and appropriate;		2 - Item needs major revision to be relevant and appropriate;	
Judge 2	4 - Relevant and appropriate item.		4 - Relevant and appropriate item.	
Judge 3	4 - Relevant and appropriate item.		4 - Relevant and appropriate item.	
Judge 4	4 - Relevant and appropriate item.		4 - Relevant and appropriate item.	
Judge 5	4 - Relevant and appropriate item.		4 - Relevant and appropriate item.	
Judge 6	4 - Relevant and appropriate item.		4 - Relevant and appropriate item.	
Judge 7	4 - Relevant and appropriate item.		4 - Relevant and appropriate item.	
Judge 8	3 - Item needs minor revision to be relevant and appropriate	complex	4 - Relevant and appropriate item.	
Judge 9	3 - Item needs minor revision to be relevant and appropriate		2 - Item needs major revision to be relevant and appropriate;	
Judge 10	4 - Relevant and appropriate item.		4 - Relevant and appropriate item.	
Judge 11	4 - Relevant and appropriate item.		4 - Relevant and appropriate item.	
Judge 12	2 - Item needs major revision to be relevant and appropriate;	If it's an open question, it will be difficult for the evaluator to define the effectiveness of digital health. If it's a closed question, I suggest separating it into categories for each item mentioned in the statement.	2 - Item needs major revision to be relevant and appropriate;	How to measure expansion?
Judge 13	4 - Relevant and appropriate item.		4 - Relevant and appropriate item.	
Judge 14	4 - Relevant and appropriate item.		4 - Relevant and appropriate item.	
Judge 15	4 - Relevant and appropriate item.		4 - Relevant and appropriate item.	
Judge 16	4 - Relevant and appropriate item.		4 - Relevant and appropriate item.	
Judge 17	4 - Relevant and appropriate item.		4 - Relevant and appropriate item.	

Database of the 1st Delphi validation round of "QualiAPS digital - Brazil"

JUDGE	(MP3) Expansion, interconnectivity and intersectoral interlocution of digital technologies to other levels of the Health Care Networks.	Suggestion/Comment	(MP4) Increased economic efficiency (maximization of resources with social well-being) resulting from the adequate allocation of resources to digital health in PHC in the promotion of equity.	Suggestion/Comment
Judge 1	2 - Item needs major revision to be relevant and appropriate;		2 - Item needs major revision to be relevant and appropriate;	
Judge 2	3 - Item needs minor revision to be relevant and appropriate	Suggestion to include interoperability	4 - Relevant and appropriate item.	
Judge 3	4 - Relevant and appropriate item.		4 - Relevant and appropriate item.	
Judge 4	4 - Relevant and appropriate item.		4 - Relevant and appropriate item.	
Judge 5	4 - Relevant and appropriate item.		4 - Relevant and appropriate item.	
Judge 6	4 - Relevant and appropriate item.		4 - Relevant and appropriate item.	
Judge 7	4 - Relevant and appropriate item.		4 - Relevant and appropriate item.	
Judge 8	3 - Item needs minor revision to be relevant and appropriate	how to measure?	3 - Item needs minor revision to be relevant and appropriate	
Judge 9	2 - Item needs major revision to be relevant and appropriate;		3 - Item needs minor revision to be relevant and appropriate	
Judge 10	4 - Relevant and appropriate item.		4 - Relevant and appropriate item.	
Judge 11	4 - Relevant and appropriate item.		4 - Relevant and appropriate item.	
Judge 12	2 - Item needs major revision to be relevant and appropriate;	If it's an open question, it will be difficult for the evaluator to compile the answers and classify the quality. If it's a closed question, I suggest separating it into categories for each item mentioned in the statement.	2 - Item needs major revision to be relevant and appropriate;	If it's an open question, it will be difficult for the evaluator to compile the answers and rate the quality. If it's a closed question, I suggest including items that can measure economic efficiency.
Judge 13	4 - Relevant and appropriate item.		4 - Relevant and appropriate item.	
Judge 14	4 - Relevant and appropriate item.		4 - Relevant and appropriate item.	
Judge 15	4 - Relevant and appropriate item.		4 - Relevant and appropriate item.	
Judge 16	4 - Relevant and appropriate item.		4 - Relevant and appropriate item.	
Judge 17	4 - Relevant and appropriate item.		4 - Relevant and appropriate item.	
