# Supplementary material for: Preparation and validation of the instrument “QualiAPS digital—Brazil” for assessing digital health care in primary health care: a required tool
Source: Front Public Health. 2024 Jul 16;12:1304148. doi: 10.3389/fpubh.2024.1304148 (PMC11286592; doi:10.3389/fpubh.2024.1304148)
Supplement: Supplementary file 2 [file Data_Sheet_2.PDF]

Database from the 2nd Delphi validation round of "QualiAPS digital - Brazil"

JUDGE	1. Number of Family Health Teams (e-FH or e-PHC) that use/used remote care actions in the unit/household/community:	Suggestion or comment	2. Number of professionals who use/used remote care in the unit/home/community:	Suggestion or comment
Judge 1	4 - Relevant and appropriate item.		4 - Relevant and appropriate item.	
Judge 2	3 - Item needs minor revision to be relevant and appropriate	I would leave the verb only in the present tense (they use), if they choose to keep the verb in the past tense (they used) I suggest adding another open question, namely: what is the reason for not using remote care actions...? This would make the item more relevant.	3 - Item needs minor revision to be relevant and appropriate	Specify the item further, namely: "Number of professionals in the Family Health Teams (e-FH ou e-PHC)..."
Judge 3	4 - Relevant and appropriate item.		4 - Relevant and appropriate item.	
Judge 4	4 - Relevant and appropriate item.		4 - Relevant and appropriate item.	
Judge 5	3 - Item needs minor revision to be relevant and appropriate	Rate or Proportion would be more appropriate. An indication of period would also be relevant depending on how often the survey is carried out (e.g. "in the last month").	3 - Item needs minor revision to be relevant and appropriate	Rate or Proportion would be more appropriate. An indication of period would also be relevant depending on how often the survey is carried out (e.g. "in the last month").
Judge 6	4 - Relevant and appropriate item.		4 - Relevant and appropriate item.	
Judge 7	4 - Relevant and appropriate item.		4 - Relevant and appropriate item.	
Judge 8	4 - Relevant and appropriate item.		4 - Relevant and appropriate item.	
Judge 9	DID NOT RESPOND TO ROUND 2	DID NOT RESPOND TO ROUND 2	DID NOT RESPOND TO ROUND 2	DID NOT RESPOND TO ROUND 2
Judge 10	DID NOT RESPOND TO ROUND 2	DID NOT RESPOND TO ROUND 2	DID NOT RESPOND TO ROUND 2	DID NOT RESPOND TO ROUND 2
Judge 11	4 - Relevant and appropriate item.		4 - Relevant and appropriate item.	
Judge 12	4 - Relevant and appropriate item.	There must be a definition of the verb tense.	4 - Relevant and appropriate item.	There must be a definition of the verb tense.
Judge 13	4 - Relevant and appropriate item.		4 - Relevant and appropriate item.	
Judge 14	3 - Item needs minor revision to be relevant and appropriate	It's interesting to think about cases that could use remote care in PHC.	3 - Item needs minor revision to be relevant and appropriate	It's interesting to think about cases that could use remote care in PHC.
Judge 15	4 - Relevant and appropriate item.		4 - Relevant and appropriate item.	
Judge 16	4 - Relevant and appropriate item.		4 - Relevant and appropriate item.	
Judge 17	3 - Item needs minor revision to be relevant and appropriate	I suggest specifying the type of remote care (teleconsultation, teleinterconsultation, teleguidance, etc.). In this way, it will be possible to know what type of remote care was provided by the team.	3 - Item needs minor revision to be relevant and appropriate	I suggest specifying the type of remote care (teleconsultation, teleinterconsultation, teleguidance, etc.). In this way, it will be possible to know what type of remote care was provided by the team.

Database of the 2nd Delphi validation round of "QualiAPS digital - Brazil"

JUDGE	3. Categories of professionals (health, IT technicians, coordinators) involved in digital health actions at the unit, district or central level:	Suggestion or comment	4. Adaptation of the physical and technological infrastructure of health units to the multiple demands (in person and remote):	Suggestion or comment
Judge 1	4 - Relevant and appropriate item.		4 - Relevant and appropriate item.	
Judge 2	3 - Item needs minor revision to be relevant and appropriate	Remove "coordinators". It's not clear what coordination refers to.	3 - Item needs minor revision to be relevant and appropriate	Specify which technology you are referring to.
Judge 3	4 - Relevant and appropriate item.		4 - Relevant and appropriate item.	
Judge 4	4 - Relevant and appropriate item.		4 - Relevant and appropriate item.	
Judge 5	4 - Relevant and appropriate item.		4 - Relevant and appropriate item.	
Judge 6	4 - Relevant and appropriate item.		4 - Relevant and appropriate item.	Just one question: isn't it important to differentiate between the suitability of the physical and technological infrastructure? One can be appropriate without the other necessarily being so.
Judge 7	4 - Relevant and appropriate item.		4 - Relevant and appropriate item.	
Judge 8	4 - Relevant and appropriate item.		3 - Item needs minor revision to be relevant and appropriate	differentiate between hardware, applications, video camera, internet access
Judge 9	DID NOT RESPOND TO ROUND 2	DID NOT RESPOND TO ROUND 2	DID NOT RESPOND TO ROUND 2	DID NOT RESPOND TO ROUND 2
Judge 10	DID NOT RESPOND TO ROUND 2	DID NOT RESPOND TO ROUND 2	DID NOT RESPOND TO ROUND 2	DID NOT RESPOND TO ROUND 2
Judge 11	4 - Relevant and appropriate item.		4 - Relevant and appropriate item.	
Judge 12	3 - Item needs minor revision to be relevant and appropriate	Specify the professional categories.	3 - Item needs minor revision to be relevant and appropriate	Specify what type of adaptation has been carried out.
Judge 13	4 - Relevant and appropriate item.		4 - Relevant and appropriate item.	
Judge 14	4 - Relevant and appropriate item.		4 - Relevant and appropriate item.	
Judge 15	4 - Relevant and appropriate item.		4 - Relevant and appropriate item.	I think it's relevant, but it's important to think about what's appropriate and who it's appropriate for.
Judge 16	4 - Relevant and appropriate item.		4 - Relevant and appropriate item.	
Judge 17	4 - Relevant and appropriate item.		4 - Relevant and appropriate item.	

Database of the 2nd Delphi validation round of "QualiAPS digital - Brazil"

JUDGE	5. Availability and quality of the internet in Health Units	Suggestion or comment	6. Interoperability between information systems (e-Sus and municipal systems) and devices/systems used for remote care:	Suggestion or comment	7. Quality of the system (e-SUS/PeC or municipal management's own systems):<br>Perception of the system's response time (fast/slow) Robustness (no crashes or execution problems) Usability (easy or requires prior training) Availability of tutorials/manuals (yes/no) Does the system have the functionalities needed for remote care? (yes/no)<br>Reporting (yes/no)	Suggestion or comment
Judge 1	4 - Relevant and appropriate item.		4 - Relevant and appropriate item.		4 - Relevant and appropriate item.	
Judge 2	4 - Relevant and appropriate item.		4 - Relevant and appropriate item.		4 - Relevant and appropriate item.	
Judge 3	4 - Relevant and appropriate item.		4 - Relevant and appropriate item.		4 - Relevant and appropriate item.	
Judge 4	4 - Relevant and appropriate item.		4 - Relevant and appropriate item.		4 - Relevant and appropriate item.	
Judge 5	4 - Relevant and appropriate item.		4 - Relevant and appropriate item.		4 - Relevant and appropriate item.	
Judge 6	4 - Relevant and appropriate item.		4 - Relevant and appropriate item.		4 - Relevant and appropriate item.	
Judge 7	4 - Relevant and appropriate item.		4 - Relevant and appropriate item.		4 - Relevant and appropriate item.	
Judge 8	4 - Relevant and appropriate item.		3 - Item needs minor revision to be relevant and appropriate	differentiating how integration is carried out	4 - Relevant and appropriate item.	
Judge 9	DID NOT RESPOND TO ROUND 2	DID NOT RESPOND TO ROUND 2	DID NOT RESPOND TO ROUND 2	DID NOT RESPOND TO ROUND 2	DID NOT RESPOND TO ROUND 2	DID NOT RESPOND TO ROUND 2
Judge 10	DID NOT RESPOND TO ROUND 2	DID NOT RESPOND TO ROUND 2	DID NOT RESPOND TO ROUND 2	DID NOT RESPOND TO ROUND 2	DID NOT RESPOND TO ROUND 2	DID NOT RESPOND TO ROUND 2
Judge 11	4 - Relevant and appropriate item.		4 - Relevant and appropriate item.		4 - Relevant and appropriate item.	
Judge 12	4 - Relevant and appropriate item.		4 - Relevant and appropriate item.		4 - Relevant and appropriate item.	
Judge 13	4 - Relevant and appropriate item.		4 - Relevant and appropriate item.		3 - Item needs minor revision to be relevant and appropriate	In the item related to the quality of the system, a topic could be inserted about guaranteeing the privacy of information for and during access.
Judge 14	4 - Relevant and appropriate item.		4 - Relevant and appropriate item.		4 - Relevant and appropriate item.	
Judge 15	4 - Relevant and appropriate item.		4 - Relevant and appropriate item.		4 - Relevant and appropriate item.	
Judge 16	4 - Relevant and appropriate item.		4 - Relevant and appropriate item.		4 - Relevant and appropriate item.	For the questions so far, I suggest a spelling check so that everyone has the same verb tense and formatting in their writing.
Judge 17	4 - Relevant and appropriate item.		4 - Relevant and appropriate item.		4 - Relevant and appropriate item.	
