# Supplementary material for: Preparation and validation of the instrument “QualiAPS digital—Brazil” for assessing digital health care in primary health care: a required tool
Source: Front Public Health. 2024 Jul 16;12:1304148. doi: 10.3389/fpubh.2024.1304148 (PMC11286592; doi:10.3389/fpubh.2024.1304148)
Supplement: Supplementary file 3 [file Data_Sheet_3.PDF]

VALIDATION OF THE QUALI APS DIGITAL – BRAZIL INSTRUMENT FOR ASSESSING THE QUALITY OF CARE IN DIGITAL HEALTH IN PRIMARY HEALTH CARE

Authors: Renan Cabral de Figueirêdo, Ísis de Siqueira Silva, Aguinaldo José de Araújo, Cícera Renata Diniz Vieira Silva, Cláudia Santos Martiniano, Ewerton William Gomes Brito, Pedro Bezerra Xavier, Severina Alice da Costa Uchôa.

INSTRUMENT 1: Questionnaire/form for PHC physicians and nurses

CHARACTERIZATION OF THE SAMPLE:

1. What type of health unit do you work in?

( ) Basic Health Unit/BHU

( ) Family Health Unit/FHU

2. What is the name of your Unit?

____

3. What is your relationship with the Municipal Health Secretary (MHS)?

( ) statutory ( ) contracted ( ) more doctors program

( ) other

4. What is your age?

( ) 20 - 30 ( ) 31 - 40 ( ) 41 - 50

( ) 51 - 60 ( ) Over 60 - elderly people

5. What is your educational background? ( ) Doctor ( ) Nurse

6. Year completed? ____

7. Do you have a postgraduate degree? ( ) Yes ( ) No

8. If yes, which postgraduate course (s)? ______________________________

____

____

9. Have you taken any courses or training in the use of digital health tools?

( ) Yes ( ) No

10. If so, which one(s)? ____

____

____

Note: The abbreviation of the indicators by letters and numbers corresponds to their identification in the "QualiAPS digital - Brazil" matrix (available at: https://doi.org/10.6084/m9.figshare.22549837.v5).

THEME 1: DIGITAL INFRASTRUCTURE IN HEALTHCARE FACILITIES

COMPONENT: STRUCTURE

DIMENSION: RESOURCES (R)

Indicator: R8. Access to and quality of equipment and tools for operationalizing digital health in Primary Health Care (PHC).

1. Does your unit have computer equipment?

( ) computers ( ) notebooks ( ) tablets

( ) wireless routers ( ) external hard drive ( ) network servers

( ) routers ( ) printer ( ) webcan

( ) microphone ( ) speaker ( ) other, please specify: ________ _______________

____

( ) There is no equipment in my unit

2. In relation to the quality of the computer equipment, how do you rate the response time when carrying out the tasks requested?

( ) Fast ( ) Slow ( ) Don't know

3. What about functionality, are there any crashes?

( ) Yes ( ) No ( ) Don't know

4. What about usability, are they easy to use?

( ) Yes ( ) No ( ) Don't know

5. Regarding the information system, does your health unit use it?

( ) e-SUS System/ Electronic Citizen Record (ECR) - PHC

( ) Another of the municipality's own computerized systems to replace ECR - PHC

( ) There is no information system in the unit

Indicator: R9. Internet availability and quality in the Health Unit.

6. Does the health unit have internet access for professionals?

( ) Yes ( ) No ( ) Don't know

Note: The abbreviation of the indicators by letters and numbers corresponds to their identification in the "QualiAPS digital - Brazil" matrix (available at: https://doi.org/10.6084/m9.figshare.22549837.v5).

7. What is your perception of the quality of the internet?

( ) Good ( ) Good ( ) Bad

( ) Very bad ( ) Don't know

Indicator: R11. System quality (e-SUS/ECR or municipal management's own systems).

8. Regarding the quality of e-SUS and ECR, how do you rate its response time, if it is used in your unit?

( ) Fast ( ) Slow ( ) Don't know

( ) Not applicable

9. Does the system crash?

( ) Yes ( ) No ( ) Don't know

10. Is the system easy to use?

( ) Yes ( ) No ( ) Don't know

THEME 2: TEAM WORK PROCESSES

Indicator: R2. Number of Primary Health Care/Family Health Unit teams that have implemented a digital health project, program or set of actions.

11. Has a digital health project or set of actions been implemented in your health unit by the PHC health teams?

( ) Yes ( ) No ( ) Don't know

12. Considering the statement that there have been health actions by digital means, what is the timing?

( ) Before the COVID 19 pandemic

( ) During the period of social distancing and temporary closure of health services in the COVID 19 pandemic (2020 to the first half of 2022)

( ) From the second half of 2022

( ) Don't know

COMPONENT: PROCESSES

DIMENSION: TECHNICAL (T)

Note: The abbreviation of the indicators by letters and numbers corresponds to their identification in the "QualiAPS digital - Brazil" matrix (available at: https://doi.org/10.6084/m9.figshare.22549837.v5).

Indicator: T1. Digital health actions carried out by Family Health/Primary Health Care teams in remote care.

13. What specific (individual and collective) digital actions (in addition to the use of the ECR) have been carried out by your unit's e-HF/PHC aimed at remote care during the critical period of the COVID-19 pandemic (2020 - 1st half of 2022)?

( ) Guidance and/or remote care for COVID-19 cases

( ) Telecare/Teleconsultation for COVID 19

( ) Telecare/Teleconsultation for non-COVID cases 19

( ) Continuity of treatment and follow-up of cases of Chronic Non-Communicable Diseases

( ) Disease prevention actions. Specify:______________________________________

____

( ) Health promotion actions. Specify: _______________________________________

____

( ) Other, please specify: ____

( ) Don't know

14. What is the target audience?

( ) Children ( ) Teenagers ( ) Adults

( ) Women ( ) Men ( ) Elderly

( ) Other, please specify: ____

( ) Not applicable

15. From the second half of 2022 (after the critical period of the pandemic), will these digital health actions continue?

( ) Yes ( ) No ( ) Don't know

16. If so, until when? ____

17. Currently (2023), what individual or collective digital health actions are carried out?

( ) Guidance and/or remote care for COVID-19 cases

( ) Telecare/Teleconsultation for COVID 19

Note: The abbreviation of the indicators by letters and numbers corresponds to their identification in the "QualiAPS digital - Brazil" matrix (available at: https://doi.org/10.6084/m9.figshare.22549837.v5).

( ) Telecare/Teleconsultation for non-COVID cases 19

( ) Continuity of treatment and follow-up of cases of Chronic Non-Communicable Diseases

( ) Disease prevention actions. Specify:_______________________________________

____

( ) Health promotion actions. Specify: ____

____

( ) Other, please specify: ____

( ) Don't know

( ) No digital health actions are carried out

18. What is the target audience?

( ) Children ( ) Teenagers ( ) Adults

( ) Women ( ) Men ( ) Elderly

( ) Other, please specify: ____

( ) Not applicable

COMPONENT: STRUCTURE

DIMENSION: RESOURCES (R)

Indicator: R7. Adequacy of the physical and technological infrastructure of health units to meet multiple demands.

19. Has the BHU's IT equipment (computers, internet, tablets, routers, printer, webcan, microphone, speaker, other) been adapted to receive face-to-face and remote demands?

( ) Yes ( ) No ( ) Don't know

( ) Not applicable

20. Has the physical infrastructure of the BHU been adapted to receive face-to-face and remote demands?

( ) Yes ( ) No ( ) Don't know

( ) Not applicable

Indicator: R2. Number of managers who have implemented a project, program or set of digital health actions in PHC.

Note: The abbreviation of the indicators by letters and numbers corresponds to their identification in the "QualiAPS digital - Brazil" matrix (available at: https://doi.org/10.6084/m9.figshare.22549837.v5).

21. Which body is responsible for digital health actions in your unit?

( ) Municipal Health Secretary - MHS

( ) Partnership with the State Health Secretary

( ) Basic Health Unit - BHU

( ) Owned by the Family Health Unit -FHU

( ) Owned by the health team

( ) Partnership with universities or research institutions - please specify: __________

____

Indicator: R5. Categories of professionals involved in remote care at the unit, district or central level.

22. Which professional categories (health, IT, management) are involved in digital health actions in PHC?

(You can check more than one option)

( ) Doctor ( ) Nurse ( ) Dentist

( ) Nursing technician/assistant ( ) Oral health technician/assistant

( ) Community Health Workers ( ) Information Technology Professional

( ) BHU manager ( ) District manager ( ) PHC manager

( ) Other ( ) Don't know

Indicator: R3. Number of Family Health/Primary Health Care teams that use/used remote care in PHC.

23. If so, how many PHC teams were involved in digital health actions in your health unit?

( ) Only 1 ( ) 2 ( ) 3

( ) Above 3 ( ) Don't know

Indicator: R.10 Digital tools used in remote care.

24. If a digital action took place in your unit, which digital Information and Communication Technology (ICT) tool(s) were used? You can check one or more alternatives

( ) Phone calls ( ) Videos ( ) SMS message

( ) social media ( ) portals

( ) cloud computing, i.e. data processing on the internet

Note: The abbreviation of the indicators by letters and numbers corresponds to their identification in the "QualiAPS digital - Brazil" matrix (available at: https://doi.org/10.6084/m9.figshare.22549837.v5).

( ) other, please specify:____

( ) I don't know

Indicator: R11. System quality (e-SUS/ECR or municipal management's own systems).

25. Do you think that the e- SUS /ECR information system already used for face-to-face care was adequate for remote care?

( ) Yes ( ) No ( ) Don't know

( ) Not applicable

Indicator: R14. Existence and adequacy of protocols, guidelines and regulations for the organization of digital health actions.

26. Are there protocols, guidelines or regulations for organizing remote care actions?
( ) Yes ( ) No ( ) Don't know

27. In your opinion, are they adequate?
( ) Yes ( ) No ( )Don't know
( ) Not applicable.

COMPONENT: RESULTS

DIMENSION: MEDIUM TERM RESULTS (MT)

Indicator: MT1. Effectiveness (relevance and appropriateness of technology choices in relation to health problems) and resoluteness of digital health.

28. Were the choices of remote care actions relevant to the health problems faced?

( ) Always ( ) Most of the time ( ) Sometimes

( ) A few times ( ) Not relevant ( ) Not applicable

29. What about the digital tools used, were they adequate?

( ) Always ( ) Most of the time ( ) Sometimes

( ) Not very often ( ) Not adequate ( ) Not applicable

30. Is digital health effective?

( ) Yes ( ) No ( ) Don't know

Note: The abbreviation of the indicators by letters and numbers corresponds to their identification in the "QualiAPS digital - Brazil" matrix (available at: https://doi.org/10.6084/m9.figshare.22549837.v5).

INSTRUMENT 2: Interview script for Primary Health Care (PHC) physicians and nurses

SAMPLE CHARACTERIZATION:

1. What type of health unit do you work for?

( ) Basic Health Unit /BHU

( ) Family Health Unit/ FHU

2. What is the name of your Unit?

____

3. What is your link with Municipal Health Secretary (MHS)?

( ) statutory ( ) contracted ( ) more doctors program

( ) other

4. What is your age range?

( ) 20 – 30 ( ) 31 – 40 ( ) 41 – 50

( ) 51 - 60 ( ) Over 60 years - elderly people

5. What is your background? ( ) Doctor ( ) Nurse

6. Year of completion?_

____

7. Do you have a graduate degree? ( ) Yes ( ) No

8. If so, which postgraduate degree (s)? ____

____

____

9. Did you take any course or training on the use of digital tools in health?

( ) Yes ( ) No

10. If so, which one (s)? ____

____

____

COMPONENT: PROCESSES

DIMENSION: TECHNIQUE (T)

Indicator: T2. Strategies, programs and projects in digital health developed in health units.

Let's talk about the period of the COVID 19 pandemic

call center

Note: The abbreviation of the indicators by letters and numbers corresponds to their identification in the "QualiAPS digital - Brazil" matrix (available at: https://doi.org/10.6084/m9.figshare.22549837.v5).

1. Did the health unit offer any health care actions through digital means?

2. If yes, talk a little about which health problems were remote care offered. For example: teleservice/teleconsultation, only for suspected cases of COVID or not? To control chronic diseases (hypertension, diabetes, psychic suffering, among others)? Promotion and prevention actions?

3. What are the digital means or tools used?

4. Which professionals are involved? And the target audience?

5. How would you briefly describe the call center

6. Were the calls recorded in the Eletronic Citizen Record (ECR)? If so, describe how the registration was processed.

7. Were data from these calls sent to MHS in a way other than ECR and e-SUS?

Monitoring

8. Were specific digital tools used to monitor COVID 19 or did you use a compulsory notification form?

Now let's talk about the period **after the return of face-to-face assistance with vaccination of the population (2022)**

9. Did the unit maintain any remote care and/or develop any other digital actions?

Indicator: T3. Qualification of health professionals to expand use among professionals and users.

10. Were health professionals qualified to apply technological resources aimed at expanding use among professionals and users?

DIMENSION: ORGANIZATIONAL (O)

Indicator: O1. Offer of actions in digital health aligned with the attributes of PHC.

11- How do you evaluate the COVID 19 call center regarding these points:

11.1 Do you think that the population's access to assistance has improved? Why?

11.2 Health care, prevention and promotion actions were articulated (comprehensiveness)

11.3 The call center was aimed at individual and/or family care

11.4 Do you think the call center was carried out in order to consider the familiarity of uses with digital media, their forms of language, understanding?

12- We are going to evaluate these same points for non-COVID call centers

12.1. Do you think that the population's access to care has improved? Why?

12.2 Care, prevention and health promotion actions were articulated (comprehensiveness)

Note: The abbreviation of the indicators by letters and numbers corresponds to their identification in the "QualiAPS digital - Brazil" matrix (available at: https://doi.org/10.6084/m9.figshare.22549837.v5).

12.3 The call center was aimed at individual and/or family care

12.4 Do you think the call center was carried out in order to consider the familiarity of uses with digital media, their forms of language, understanding?

Indicator: O2. Health surveillance actions through Information and Communication Technologies in the territories.

13. Is there development of actions in health surveillance (EPIDEMIOLOGICAL/HEALTH/ENVIRONMENT) through Information and Communication Technology (ICTs) in the territories?

If yes, talk about this action.

Indicator: O3. Interoperability between information systems (e-SUS and municipal systems) with devices/systems used in remote care.

14. What digital tools were used to carry out remote actions?

15. How do you evaluate these tools? Do they suit the health problems for which they were applied? What are the positives and negatives (speed, timely reporting, crashes)?

16. Do these tools allow interoperability (the ability of different systems to work together) with the ECR and e -SUS? Or with other proprietary systems

Indicator: O5. Management incentive actions for innovations in management, assistance and educational technologies.

17. Did actions via digital means, whether carried out at the BHU or in the assigned area of the unit, community or even at home, receive MHS incentives such as the provision of computer equipment, internet, support from an IT professional, technical assistance?

18. Has Human Resources been trained to use technological tools?

DIMENSION: RELATIONAL (RE)

Indicator: RE2. Reinforcement of welcoming and bonding with users, through the technology interface.

19. How is reception carried out in remote activities?

20. What strategies for establishing or maintaining the bond are carried out in remote activities?

Indicator: RE3. Use of technology interface with impact on improving teamwork.

21. Did the use of the technological interface impact teamwork? In what way?

Note: The abbreviation of the indicators by letters and numbers corresponds to their identification in the "QualiAPS digital - Brazil" matrix (available at: https://doi.org/10.6084/m9.figshare.22549837.v5).

COMPONENT: RESULTS

DIMENSION: MEDIUM TERM RESULTS (MT)

Indicator: MT1. Effectiveness of digital health (pertinence of technology choices in relation to health problems).

22. Talk a little about the pertinence of the actions developed in relation to the health problems faced.

23. Now about the digital tools used.

COMPONENT: RESULTS

DIMENSION: SHORT TERM RESULTS (ST)

Indicator: ST1. Expansion of the offer of care with support for lines of care.

24. In general, do you consider that the use of digital technologies has increased access? Just regarding COVID 19? And or also to other types of remote care offered?

25. Were remote assistance adequate to establish a line of care for users? Why?

Indicator: ST2. More accessible health care and maintenance of supply.

26. Based on your experience, do you consider that care through Information and Communication Technology is accessible to BHU professionals? Why?

27. And for users?

28. Was it possible to maintain continuity of care (longitudinality) with remote care? Why?

Indicator: ST6. Protagonism of the actors involved: satisfaction, confidence in technologies and practices in regulatory and ethical standards.

29. As a health professional, did you feel comfortable, that is, satisfied with the use of digital tools? Why?

30. From your experience, do you have confidence in the resolution of the health problem when you resorted to remote care? Explain

31. What is your opinion about data security and the use of digital tools in the context you used?

32. What about ethical aspects?

Note: The abbreviation of the indicators by letters and numbers corresponds to their identification in the "QualiAPS digital - Brazil" matrix (available at: https://doi.org/10.6084/m9.figshare.22549837.v5).

INSTRUMENT 3: Interview script for Information Technology (IT) professionals.

SAMPLE CHARACTERIZATION:

1. What is your age group?

( ) 20 – 30 ( ) 31 – 40 ( ) 41 – 50

( ) 51 - 60 ( ) Over 60 years - elderly people

2. Training in Information Technology (IT)?

Course: ____

Conclusion year:____

3. Do you have training/training in health?

Course: ____

Conclusion year: ____

4. Link with the institution?

( ) Statutory ( ) Hired ( ) Outsourced

( ) Collaborator of the institution ( ) Other

5. Location? ____

COMPONENT: STRUCTURE

DIMENSION: RESOURCES (R)

Indicator: R6. Information and Communication Technology professionals involved/offering support in digital health actions in Primary Health Care (PHC).

1. Do IT professionals support health teams to offer actions using ICTs in PHC?

2. How many professionals make up the IT team and what is its composition (technical assistance, developers, analysts, digital communicators, designers, etc.)?

3. Do the IT professionals have any training/training courses in the health area?

Indicator: R12. Existence of a technical assistance service for digital tools used in remote care in PHC.

4. Is technical assistance provided for digital tools used in remote care in PHC?

If so, preventive and/or corrective?

Own, outsourced by Municipal Health Secretary (MHS) or from partner institutions?

Note: The abbreviation of the indicators by letters and numbers corresponds to their identification in the "QualiAPS digital - Brazil" matrix (available at: https://doi.org/10.6084/m9.figshare.22549837.v5).

COMPONENT: PROCESSES

DIMENSION: TECHNIQUE (T)

Indicator: T1. Digital health actions carried out in remote care in PHC.

5. Have you participated or do you participate in any action or IT project aimed at PHC in the municipality?

If yes, which one and when?

If not (study exclusion criterion, close and look for another subject).

Could you provide a document describing this action or project?

6. What was your role/function in this action/project?
7. What other IT and healthcare professionals were involved?

Talk a little more about these actions:

8. Are/were these actions/projects focused only on COVID 19 and/or other health problems?

If for other problems, which ones?

9. Were they one-off or continuous actions?
10. Were they actions aimed at management and/or assistance to individual health, self-care or actions aimed at the collective, such as case monitoring, epidemiological surveillance, health education, others?
11. Did they continue after suspension of social distancing and full return of health services (2022-2023)?
12. Could you mention which Information and Communication Technologies (ICTs) were used?
13. Talk a little about the relevance of the tools used in relation to the health problems faced?
14. Did the IT team train the healthcare professional staff?

If you offered training, could you make this material available?

15. Were the health teams provided with didactic material, tutorials on the use of ICTs ?

Indicator: T4. Technical support for the use of technologies with guaranteed security and protection of personal data.

16. Did the IT team offer technical support to health professionals who used ICTs (such as guidance, clarification of doubts, manuals and/or other types of support) in PHC?

17. Are/were there data security guarantees?

DIMENSION: ORGANIZATIONAL (O)

Note: The abbreviation of the indicators by letters and numbers corresponds to their identification in the "QualiAPS digital - Brazil" matrix (available at: https://doi.org/10.6084/m9.figshare.22549837.v5).

Indicator: O3. Interoperability between information systems (e-Sus and municipal systems) with devices/systems used in remote care

18. The digital tools that were/are used by PHC health teams in remote care are/were integrated* into the Ministry of Health's information system, such as, for example, health information systems (e-SUS) Electronic Citizen Record (ECR) or other municipal information systems?

*Interoperability is the ability to make the connection between systems, whereas integration is interoperability in practice.

19. If so, how do you rate some aspects of system performance such as response time*, robustness**, usability***, availability****?

*Response time refers to the time it takes the system to respond to a user request. This property is related to system performance and can involve both the processing speed of requested actions and the response time of the user interface. Evaluating response time is important to ensure the system is agile and responsive to user interactions.

**Robustness refers to the system's ability to deal with different conditions and situations, maintaining its proper functionality and performance. A robust system is able to handle errors, exceptions and failures without significant interruptions. Assessing robustness involves checking whether the system is able to recover from failures, handle invalid input, and ensure data integrity.

*** Usability refers to the ease of use of the system and the user's experience when interacting with it. An intuitive, well-designed and user-friendly user interface makes the system easy to use and makes the user experience more enjoyable. Evaluating usability involves checking the clarity and organization of the interface, consistency of interactions, ease of learning and efficiency in using the system.

**** Availability refers to the ability of the system to be available and accessible to users when needed. An available system is up and running and accessible during the planned or agreed time. Assessing availability involves verifying that the system is functioning correctly, that there are no frequent outages, that backup and recovery mechanisms are in place in case of failures, and that downtime is minimized.

20. In general, how do you evaluate the functionality* of the system used (e-SUS or other)?

*****Functionality includes data collection, storage, processing and analysis, information retrieval, communication and collaboration, process automation, decision support, among others.

COMPONENT: RESULTS

DIMENSION: MEDIUM TERM RESULTS (MT)

Indicator: MT2. Expansion of computerization in the APS.

21. In your experience with ICTs in PHC in the municipality, how do you evaluate the advances and challenges of its application?

INSTRUMENT 4: Script for a focal group with Community Health Workers (CHWs).

SAMPLE CHARACTERIZATION:

1. What type of health unit do you work for?

( ) Basic Health Unit /BHU

( ) Family Health Unit/ FHU

2. What is the name of your Unit?

____

3. What is your link with Municipal Health Secretary (MHS)?

( ) statutory ( ) contracted ( ) other

4. What is your age range?

( ) 20 – 30 ( ) 31 – 40 ( ) 41 – 50

( ) 51 - 60 ( ) Over 60 years - elderly people

5. Do you have a Community Health Workers technical course?

( ) Yes ( ) No

6. Did you take any course or training on the use of digital tools in health?

( ) Yes ( ) No

7. If so, which one (s)? ____

____

____

THEME 1: DIGITAL INFRASTRUCTURE OF HEALTH CARE UNITS

COMPONENT: STRUCTURE

DIMENSION: RESOURCES (R)

Indicator: R8. Access and quality of equipment for the operationalization of digital health.

1. What computer equipment do you have access to carry out your work activities? (computers, notebooks, tablets, routers, external HD, network servers, printer, webcam, microphone, speaker...)

2. Regarding the quality of this equipment, what are the positive and negative aspects (speed, crashes, ease of use...)?

Indicator: R9. Availability and quality of the internet in the Health Unit.

3. Does the health unit have internet access for professionals?

Note: The abbreviation of the indicators by letters and numbers corresponds to their identification in the "QualiAPS digital - Brazil" matrix (available at: https://doi.org/10.6084/m9.figshare.22549837.v5).

4. How do you rate it?

Indicator: R11. System quality (e-SUS/ECR or municipal management systems).

5. As for the quality of e-SUS and Electronic Citizen Record (ECR), what are the positive and negative aspects (speed, crashes, ease of use...)?

Indicator: R5. Categories of professionals involved in remote care in Primary Health Care (PHC).

6. Were you, CHWs, involved in remote care for users during the critical period of the pandemic (2020 – 1st half of 2022)? Talk about that process.

Indicator: R10. Digital tools used in remote care.

7. If involved in digital health actions, which digital tool (s) of Information and Communication Technology (ICT) was (were) used?

(Phone calls, videos, MHS messages, messages via apps, social media, portals, cloud computing, “cloud”, i.e. data processing on the internet...)

Indicator: R7. Adequacy of the physical and technological infrastructure of the health units for the multiple demands (on-site and remote).

8. Was there any adaptation of the IT equipment (computers, internet, tablets, routers, printer, webcam, microphone, speaker, other) at the health unit to receive face-to-face and remote demands? Which?

9. Was there adequacy in the physical infrastructure of the health unit to receive face-to-face and remote demands? Which?

Indicator: R14. Existence and adequacy of protocols, guidelines and regulations for the organization of digital health actions.

10. Was any protocol, guideline or regulation made available for the organization of remote care actions? If so, could you make this material available?

11. In your opinion, are they adequate to the reality experienced?

COMPONENT: PROCESSES

DIMENSION: TECHNIQUE (T)

Indicator: T1. Digital health actions carried out by Family Health (FH)/Primary Health Care (PHC) teams in remote care.

Note: The abbreviation of the indicators by letters and numbers corresponds to their identification in the "QualiAPS digital - Brazil" matrix (available at: https://doi.org/10.6084/m9.figshare.22549837.v5).

12. What specific (individual and collective) digital actions (in addition to the use of the ECR) were carried out by the e-FH/PHC of your unit aimed at remote care during the critical period of the COVID-19 pandemic (2020 - 1st half of 2022)?

(Guidelines and/or remote care for COVID 19 cases, teleservice/ teleconsultation for COVID 19, continuity of treatment and follow-up of cases of Chronic Noncommunicable Diseases, health promotion actions, virtual visits...).

13. As of the 2nd half of 2022, will these digital health actions continue? If yes, until when?

14. Currently (2023), are individual or collective digital health actions carried out? Which?

DIMENSION: RELATIONAL (RE)

Indicator: RE1. Professional engagement and community participation in the choice, implementation and evaluation of technologies.

15. How was the choice of tools or applications used for remote care?

16. What about the injuries and target audience assisted?

17. Has any technology assessment been carried out? As?

18. Did users participate in any of these processes? Which one (is) and in what way?

Indicator: RE2. Reinforcement of welcoming and bonding with users, through the technology interface.

19. H ow is reception carried out in remote activities?

20. What strategies for establishing or maintaining the bond are carried out in remote activities?

Indicator: RE3. Use of technology interface with impact on improving teamwork.

21. Did the use of the technological interface impact teamwork? In what way?

COMPONENT: RESULTS

DIMENSION: SHORT TERM RESULTS (ST)

Indicator: ST1. Expansion of the offer and support for lines of care.

22. In general, do you consider that the use of digital technologies has increased access? Just regarding COVID 19? And or also to other types of remote care offered?

23. Were remote assistance adequate to establish a line of care for users? Why?

Note: The abbreviation of the indicators by letters and numbers corresponds to their identification in the "QualiAPS digital - Brazil" matrix (available at: https://doi.org/10.6084/m9.figshare.22549837.v5).

Indicator: ST2. More accessible health care and maintenance of supply.

24. Based on your experience, do you consider that care through Information and Communication Technology is accessible to health unit professionals? Why?

25. And for users?

26. Was it possible to maintain continuity of care (longitudinality) with remote care? Why?

Indicator: ST6. Protagonism of the actors involved: satisfaction, confidence in technologies and practices in regulatory and ethical standards.

27. As a health professional, did you feel comfortable, that is, satisfied with the use of digital tools? Why?

28. From your experience, do you have confidence in the resolution of the health problem when you resorted to remote care? Explain

29. What is your opinion about data security and the use of digital tools in the context you used?

30. What about ethical aspects?

Note: The abbreviation of the indicators by letters and numbers corresponds to their identification in the "QualiAPS digital - Brazil" matrix (available at: https://doi.org/10.6084/m9.figshare.22549837.v5).

INSTRUMENT 5: Interview script for PHC managers.

SAMPLE CHARACTERIZATION:

1. What is your age group?

( ) 20 – 30 ( ) 31 – 40 ( ) 41 – 50

( ) 51 - 60 ( ) Over 60 years - elderly people

2. What is the current position?

( ) Health Secretary ( ) PHC Coordinator

( ) Health District Manager ( ) Health Unit Manager

3. Workplace? ____

____

4. Do you have a degree? ( ) Yes ( ) No

5. If yes, which one?

____

6. Year completed? ____

____

7. Do you have a graduate degree? ( ) Yes ( ) No

8. If so, which postgraduate degree (s)? ____

____

____

COMPONENT: STRUCTURE

DIMENSION: RESOURCES (R)

Indicator: R2. Number of managers who implemented some project, program or set of digital health actions in Primary Health Care (PHC).

1. Does the municipality participate in any program to encourage digital health through the Ministry of Health?

2. Was there any independent project, program or set of actions in digital health (remote care) implemented by management? Which one (s)?

Indicator: R1. Financial resources destined to fund actions in digital health in PHC.

3. Has the municipality received or receives financial transfers to fund digital health in PHC? Comment a little about these transfers (quantitative, percentage, temporality, applicability...)

Note: The abbreviation of the indicators by letters and numbers corresponds to their identification in the "QualiAPS digital - Brazil" matrix (available at: https://doi.org/10.6084/m9.figshare.22549837.v5).

Indicator: R5. Categories of professionals (health, information technology, management) involved in remote care at the unit, district or central level.

4. Which professional categories (health, IT, management) are involved in digital health actions in PHC?

Indicator: R3. Quantitative of Family Health (FH)/Primary Health Care teams that use/used remote care in PHC.

5. How many FH/PHC teams used remote care during the critical period of the pandemic (2020 - 1st half of 2022) and how many are still using it?

Indicator: R4. Number of professionals who use/used remote care in PHC.

6. Approximately how many professionals used remote care in PHC in the health unit, district or municipality during the critical period of the pandemic (2020-2022.1)?

7. Do these professionals continue to use remote care in PHC after returning to face-to-face activities? About how many remain?

Indicator: R12. Existence of a technical assistance service for digital tools used in remote care in PHC.

8. Is technical assistance provided for digital tools used in remote care in PHC? Preventive and/or corrective?

9. Own, outsourced by the municipal health department or partner institutions?

Indicator: R14. Protocols, guidelines and regulations for organizing digital health actions.

10. Was there adoption of protocols, guidelines or regulations for the organization of remote care actions?

11. If so, in your opinion, are they suitable?

- Could you make this material available?

Indicator: R13. Existence of partnerships (state/municipal telehealth and telemedicine centers; universities; other Information and Communication Technology centers).

12. Are there partnerships with state/municipal/university telehealth /telemedicine centers or other Information and Communication Technology centers? Which?

COMPONENT: PROCESSES

Note: The abbreviation of the indicators by letters and numbers corresponds to their identification in the "QualiAPS digital - Brazil" matrix (available at: https://doi.org/10.6084/m9.figshare.22549837.v5).

DIMENSION: TECHNICAL (T)

Indicator: T3. Qualification of managers to expand the use of digital technologies among professionals and users.

13. Was there qualification of managers for the application of technological resources aimed at expanding use among professionals and users? In what way?

DIMENSION: ORGANIZATIONAL (O)

Indicator: O4. Planning of technological solutions for Primary Health Care demands with monitoring and management of resources.

14. How was the planning for the use of digital health in PHC?

15. How are resources monitored and managed?

Indicator: O5. Management incentive actions for innovations in management, assistance and educational technologies.

16. What incentive actions does management provide for innovations in technologies, whether managerial, assistance or educational in health?

(for example: Training, infrastructure, technological resources, investments, others)

Indicator: O6. Partnerships of the Municipal Health Secretariat (MHS) with research aimed at digital solutions to problems in PHC.

17. Does MHS have partnerships with research aimed at digital solutions for health problems in PHC? Which one (s)?

COMPONENT: RESULTS

DIMENSION: SHORT TERM RESULTS (ST)

Indicator: ST3. Development and implementation of synchronous, asynchronous and monitoring technological tools, promoting management, assistance and educational actions in PHC.

18. What synchronous, asynchronous and/or monitoring technological tools contributed to the promotion of managerial, assistance and educational actions in PHC?

Indicator: ST4. Institutionalization of training programs for the inclusion of digital health aimed at PHC professionals and users.

19. Was there institutionalization of permanent education strategies for the inclusion of digital health in PHC aimed at professionals and users? Name a few:

Note: The abbreviation of the indicators by letters and numbers corresponds to their identification in the "QualiAPS digital - Brazil" matrix (available at: https://doi.org/10.6084/m9.figshare.22549837.v5).

Indicator: ST5. Active governance system connected to the needs of the services, incentives for the expansion of digital health programs, quality improvement cycles and evaluative research.

20. How is the system for monitoring service needs in the field of digital health?

21. What strategies are developed with a view to expanding digital health and service quality improvement cycles?

22. How are these services evaluated?

DIMENSION: MEDIUM TERM RESULTS (MT)

Indicator: MT3. Expansion of the interconnectivity and interlocution of digital technologies to other levels of the Health Care Networks (HCN).

23. How is communication via digital technologies from PHC to other levels of the Health Care Networks?

Indicator: MT1. Efficacy (capacity to produce improvement) in the quality of care offered and resolution of digital health.

24. Regarding effectiveness (capacity to produce improvement) in the quality of care offered, what are the positive effects of adherence by the actors involved?

25. What is your opinion about the resolution of digital health?

Indicator: MT4. Increased economic efficiency (maximization of resources with social well-being) and promotion of equity.

26. Is it possible to talk about economic efficiency (maximization of resources with social well-being) when dealing with digital health in PHC? Comment on your answer:

27. With regard to the principle of equity, how can we combat digital exclusion in health services?

Indicator: MT2. Expansion of computerization in Primary Health Care.

28. What are the advances and challenges of expanding computerization in PHC?

Note: The abbreviation of the indicators by letters and numbers corresponds to their identification in the "QualiAPS digital - Brazil" matrix (available at: https://doi.org/10.6084/m9.figshare.22549837.v5).
