# Supplementary material for: Preparation and validation of the instrument “QualiAPS digital—Brazil” for assessing digital health care in primary health care: a required tool
Source: Front Public Health. 2024 Jul 16;12:1304148. doi: 10.3389/fpubh.2024.1304148 (PMC11286592; doi:10.3389/fpubh.2024.1304148)
Supplement: Supplementary file 4 [file Data_Sheet_4.PDF]

renasf
REDE NORDESTE DE FORMAÇÃO EM SAÚDE DA FAMÍLIA

VALIDATION OF THE QUALI APS DIGITAL – BRAZIL INSTRUMENT FOR ASSESSING THE QUALITY OF CARE IN DIGITAL HEALTH IN PRIMARY HEALTH CARE

Authors: Renan Cabral de Figueirêdo, Ísis de Siqueira Silva, Aguinaldo José de Araújo, Cícera Renata Diniz Vieira Silva, Cláudia Santos Martiniano, Ewerton William Gomes Brito, Pedro Bezerra Xavier, Severina Alice da Costa Uchôa.

Instrument for Physicians and Nurses in Primary Health Care (PHC)

1. Before the COVID-19 pandemic, was any remote care action developed by e-FH/PHC? Yes/no
2. If yes, which one(s):
____
____
3. What individual and collective actions were carried out by e-FH/PHC in remote care during the critical period of the COVID-19 pandemic (2020 - 2022.1)?
4. What individual and collective actions continue to be carried out by e-FH/PHC in remote care after the critical period of the COVID-19 pandemic (from 2022.2 onwards)? If they continue...
5. What is the frequency of activities? (one-time or ongoing)
6. If ongoing, how often? (daily, weekly, bi-weekly, monthly, other _________________)
7. What categories of professionals are involved in these activities?
8. What is the target population?
9. Has the municipality joined any program for funding digital health actions? Yes/no
10. Does the municipality participate in any program to promote digital health through MH (Ministry of Health)? Yes/No
11. Was any project, program, or set of actions in digital health (remote care) independently implemented by the management? Yes/no
12. If yes, which project(s), program(s), or actions?
13. Are there management incentives for innovations in managerial, care, and educational health technologies? Yes/no
14. If yes, which ones? (Training, infrastructure, technological resources, investments, other)
____
____
15. Are there actions in health surveillance (EPIDEMIOLOGICAL/SANITARY/ENVIRONMENTAL) through ICTs in the territories? Yes/no
16. If yes, which one(s)? (epidemiological/sanitary/environmental) Specify the action:
____
17. During the critical period of the COVID-19 pandemic (2020 to 2022.1), were there any actions in health surveillance (EPIDEMIOLOGICAL/SANITARY/ENVIRONMENTAL) through ICTs in the territories? Yes/no/don't know
18. If yes, which one(s)? (epidemiological/sanitary/environmental) Specify the action:
____

19. Are the remote care actions aligned with the essential attributes (access, comprehensiveness, longitudinality, and care coordination) and derived ones (family orientation, community and cultural competence) of PHC? Yes/no
20. Are there protocols, guidelines, or regulations for organizing remote care actions? Yes/no
21. If yes, in your opinion, are they adequate? Yes/no
22. Have professionals received training to apply technological resources aiming at expanding their use among professionals and users? Yes/no
23. What strategies, programs, and/or projects for remote care were developed within and outside the physical spaces of health units?
24. Does the health unit have internet access for professionals? Yes/no
25. If yes, what is your perception of the quality of the internet? Excellent/good/poor/very poor
26. Is there interoperability between the devices used in remote care and the adopted information system? Yes/no
27. What is your perception of the response time of the information system used (e-SUS/PeC or municipal management's own systems)? Fast/slow
28. Regarding robustness (does it have execution problems)? Crashes/does not crash
29. Regarding usability? Easy/requires prior training
30. Are tutorials/manuals available? Yes/no
31. Does the system have necessary functionalities for remote care? Yes/no
32. Is it possible to generate reports? Yes/no
33. Is there technical assistance service for the digital tools used in remote care in PHC? Yes/no/don't know
34. If yes, is it preventive or corrective?

Were health professionals and the community involved in the following questions:

35. In choosing the type of tool or application used? (Example: WhatsApp, Google Meet, videos, phone calls, etc...)
Professionals ( ); Community ( ); Both ( ); None ( )
36. In addressing assisted problems
Professionals ( ); Community ( ); Both ( ); None ( )
37. In targeting actions
Professionals ( ); Community ( ); Both ( ); None ( )
38. In the frequency of actions
Professionals ( ); Community ( ); Both ( ); None ( )
39. In the type of activity
Professionals ( ); Community ( ); Both ( ); None ( )
40. In evaluating technologies
Professionals ( ); Community ( ); Both ( ); None ( )
41. Was there concern with user reception and connection through the technological interface during the critical period of the pandemic (2020 - 2022.1)? Yes/no
42. Mention strategies used for reception:
43. Mention strategies used to maintain connection:
44. Currently, how is reception handled in remote activities?
45. Did the development and use of synchronous, asynchronous, and monitoring technological tools contribute to managerial actions in PHC? Positively/negatively/did not contribute
46. Did the development and use of synchronous, asynchronous, and monitoring technological tools contribute to care actions in PHC? Positively/negatively/did not contribute

47. Did the development and use of synchronous, asynchronous, and monitoring technological tools contribute to educational actions in PHC? Positively/negatively/did not contribute
48. Were institutional digital health training programs provided to PHC professionals and users? (only for professionals, only for users, for both, not provided)
49. Is there protagonism of the involved actors with a clear definition of responsibility? Yes/no
50. Do professionals trust the technologies? Yes/no
51. Do users trust the technologies? Yes/no
52. Are you satisfied with the use of the technologies? Very satisfied, satisfied, dissatisfied, very dissatisfied.
53. Does the practice adhere to ethical precepts? Yes/no
54. Regarding the effectiveness of the quality of care offered, were there positive effects from the adherence of the involved actors? Yes/no
55. Are users satisfied? Very satisfied, satisfied, dissatisfied, very dissatisfied
56. Is digital health effective? Yes/no
57. Was there technical accuracy (appropriateness of technology choices regarding health problems)? Yes/no
58. Did digital health provide continuity and care coordination in Health Care Networks (HCN)? Yes/no
59. Is there a system for monitoring service needs to encourage digital health expansion? Yes/no/don't know
60. Is there a system for monitoring service needs to encourage quality care improvement cycles? Yes/no/don't know
61. Is there a system for monitoring service needs to encourage evaluative research? Yes/no/don't know
62. Was there an expansion of computerization in PHC (strengthening information systems with data integrity, reliability, and information consistency throughout their life cycle and operating systems)? Yes/no/don't know
63. Was there an increase in interconnectivity and intersectoral dialogue of digital technologies to other levels of Health Care Networks? Yes/no/don't know

Interview Instrument for Information Technology (IT) Professionals

1. Is there technical support for the technologies used in primary health care (PHC)? Yes/no
2. If yes, does the technical support provided ensure the security of personal data? Yes/no
3. Is there interoperability between the devices used in remote care and the adopted information system? Yes/no
4. Is there technical assistance service for the digital tools used in remote care in PHC? Yes/no
5. If yes, is it preventive or corrective?
6. How many IT technicians support remote care actions in PHC?
None, other: ___________
7. If yes, do they have any training/education courses in the healthcare field? Yes/no/don't know
8. If yes, which ones?
____

9. Has there been an expansion of computerization in PHC (strengthening information systems with data integrity, reliability, and consistency throughout their lifecycle, and operating systems)? Yes/no/don't know
10. Has there been an increase in the interconnectivity and intersectoral dialogue of digital technologies with other levels of Health Care Networks? Yes/no/don't know

Instrument for Community Health Workers (CHWs)

1. Did e-FH/PHC use remote care in PHC during the critical period of the pandemic (2020-2022.1)? Yes/no
2. What strategy/ies? (teleconsultation, teleconsultancy, telediagnosis, teleregulation, telemonitoring, teleorientation, second formative opinion, other)
3. Does e-SF/AB continue to use remote care in PHC? Yes/no
4. What strategy/ies? (teleconsultation, teleconsultancy, telediagnosis, teleregulation, telemonitoring, teleorientation, second formative opinion, other)
5. What professionals are involved in remote care at the unit, district, or central level?
Healthcare: ____
IT Technicians: ____
Management: ____
6. Before the COVID-19 pandemic, was any remote care action developed by e-FH/PHC? Yes/no
7. If yes, which one(s):
____
____
8. What individual and collective actions were carried out by e-FH/PHC in remote care during the critical period of the COVID-19 pandemic (2020-2022.1)?
9. What individual and collective actions continue to be carried out by e-FH/PHC in remote care after the critical period of the COVID-19 pandemic (from 2022.2 onwards)? If they continue...
10. What is the frequency of activities? (one-time or ongoing)
11. If ongoing, how often? (daily, weekly, bi-weekly, monthly, other _________________)
12. What categories of professionals are involved in these activities?
13. What is the target population?
14. Are there actions in health surveillance (EPIDEMIOLOGICAL/SANITARY/ENVIRONMENTAL) through ICTs in the territories? Yes/no
15. If yes, which one(s)? (epidemiological/sanitary/environmental) Specify the action: ____
16. During the critical period of the COVID-19 pandemic (2020 to 2022.1), were there any actions in health surveillance (EPIDEMIOLOGICAL/SANITARY/ENVIRONMENTAL) through ICTs in the territories? Yes/no/don't know
17. If yes, which one(s)? (epidemiological/sanitary/environmental) Specify the action: ____
18. Was there physical infrastructure adaptation of the UBS to receive face-to-face and remote demands? Yes/no
19. If yes, which one(s)?____

20. Was there technological infrastructure adaptation (computers, internet, tablets, routers, printer, webcam, microphone, speakers, other) of the UBS to receive face-to-face and remote demands? Yes/no
21. If yes, which one(s)?____

22. What digital tool(s) (ICT) is/are used in remote care?
- Phone calls, videos, text messages via apps, SMS messages, social media, portals, cloud computing, other (specify):
____
____
23. Are there protocols, guidelines, or regulations for organizing remote care actions? Yes/no
24. If yes, in your opinion, are they adequate? Yes/no
25. Are the equipment for remote care operationalization institutional? Yes/no
If yes, how do you rate the quality of the equipment:
26. What is your perception of response time? Fast/slow
27. Regarding robustness? Does it have execution problems/does not crash
28. Regarding usability? Easy/difficult with prior training
29. Does the health unit have internet access for professionals? Yes/no
30. If yes, what is your perception of the internet quality? Excellent, good, poor, very poor
31. Is there interoperability between the devices used in remote care and the adopted information system? Yes/no
32. What is your perception of the response time of the information system used (e-SUS/PeC or municipal management's own systems)? Fast/slow
33. Regarding robustness (does it have execution problems)? Crashes/does not crash
34. Regarding usability? Easy/needs prior training
35. Are tutorials/manuals available? Yes/no
36. Does the system have necessary functionalities for remote care? Yes/no
37. Is it possible to generate reports? Yes/no
38. Is there technical assistance service for the digital tools used in remote care in PHC? Yes/no/don't know
40. If yes, preventive or corrective?
Were health professionals and the community involved in the following questions:
41. In choosing the type of tool or application used? (Example: WhatsApp, Google Meet, videos, phone calls, etc...)
Professionals ( ); Community ( ); Both ( ); None ( )
42. In addressing assisted problems
Professionals ( ); Community ( ); Both ( ); None ( )
43. In targeting actions
Professionals ( ); Community ( ); Both ( ); None ( )
44. In the frequency of actions
Professionals ( ); Community ( ); Both ( ); None ( )
45. In the type of activity
Professionals ( ); Community ( ); Both ( ); None ( )
46. In evaluating technologies
Professionals ( ); Community ( ); Both ( ); None ( )

47. Was there concern with user reception and connection through the technological interface during the critical period of the pandemic (2020 - 2022.1)? Yes/no
48. Mention strategies used for reception:
49. Mention strategies used to maintain connection:
50. Currently, how is reception handled in remote activities?

51. Did the use of the technological interface impact teamwork during the critical period of the pandemic (2020 - 2022.2)? Yes/no
52. If yes, positively or negatively
53. Was there an expansion of care offerings with the use of digital health strategies? Yes/no
54. Were the practices, technologies, and instruments adopted adequate to support care lines during the critical period of the pandemic (2020 - 2022.2)? Yes/no
55. Did healthcare become more accessible with the help of ICTs? Yes/no
56. Is care continuity possible with the use of ICTs? Yes/no
57. Did the development and use of synchronous, asynchronous, and monitoring technological tools contribute to managerial actions in PHC? Positively/negatively/did not contribute
58. Did the development and use of synchronous, asynchronous, and monitoring technological tools contribute to care actions in PHC? Positively/negatively/did not contribute
59. Did the development and use of synchronous, asynchronous, and monitoring technological tools contribute to educational actions in PHC? Positively/negatively/did not contribute
60. Were institutional digital health training programs provided to professionals and users of PHC? Only for professionals, only for users, for both, not provided
61. Is there protagonism of the involved actors with a clear definition of responsibility? Yes/no
62. Do professionals have confidence in the technologies? Yes/no
63. Do users have confidence in the technologies? Yes/no
64. Are you satisfied with the use of the technologies? Very satisfied, satisfied, dissatisfied, very dissatisfied.
65. Does the practice adhere to ethical precepts? Yes/no
66. Regarding the effectiveness of the quality of care offered
67. Are users satisfied? Very satisfied, satisfied, dissatisfied, very dissatisfied
68. Is digital health resolving? Yes/no
69. Was there technical accuracy (appropriateness of technology choices regarding health problems)? Yes/no
70. Did digital health provide continuity and coordination of care in Health Care Networks (HCN)? Yes/no

Instrument for Primary Health Care Managers

1. Has the municipality adhered to any funding program for digital health actions? Yes/no
2. Does the municipality participate in any digital health incentive program through the Ministry of Health? Yes/no
3. Has the management implemented any project, program, or set of digital health actions (remote care) independently? Yes/no
4. If yes, what project(s), program(s), or actions?

5. Was there planning for the use of digital health focused on the demands of Primary Health Care (PHC)? Yes/no/Don't know
6. How is the monitoring and management of resources carried out?
7. Are there management-initiated incentive actions for innovations in managerial, care-related, and educational health technologies? Yes/no
8. If yes, what are they? (Training, infrastructure, technological resources, investments, other)
9. Has there been institutionalization of permanent education strategies for the inclusion of digital health in PHC directed at professionals and users? No/only for professionals/only for users/for both
10. Are there partnerships with state/municipal telehealth/telemedicine centers, universities, or other ICT (Information and Communication Technology) nuclei? Yes/no/don't know
11. If yes, which ones?
12. Does the Municipal Health Secretariat (MHS) have partnerships with research focused on digital solutions for health problems in PHC? Yes/no/don't know
13. If yes, which ones?
14. Which professionals are involved in remote care at the unit, district, or central level?
- Health professionals: ________________________________
- IT Technicians: ________________________________
- Management: ________________________________
15. Have professionals undergone training to apply technological resources aiming to expand their use among professionals and users? Yes/no
16. Have managers undergone training to apply technological resources aiming to expand their use among professionals and users? Yes/no
17. How many IT technicians support remote care actions in PHC?
None, other: ______________________
18. If yes, do the technician(s) have any training/course in the health area? Yes/no
19. If yes, which one? ______________________/don't know
20. Is there a monitoring system of service needs aimed at encouraging the expansion of digital health? Yes/no/don't know
21. Is there a monitoring system of service needs aimed at encouraging quality improvement cycles of care? Yes/no/don't know
22. Is there a monitoring system of service needs aimed at encouraging evaluative research? Yes/no/don't know
23. Has there been an increase in computerization in PHC (strengthening information systems with data integrity, reliability, and consistency throughout their life cycle and operating systems)? Yes/no/don't know
24. Has there been an increase in interconnectivity and intersectoral interaction of digital technologies to other levels of Health Care Networks? Yes/no/don't know
25. Has there been an increase in economic efficiency (maximization of resources with social well-being) resulting from the proper allocation of resources to digital health in PHC in promoting equity? Yes/no
26. Are there protocols, guidelines, or regulations for the organization of remote care actions? Yes/no
27. If yes, in your opinion, are they adequate? Yes/no
28. What strategies, programs, and/or projects for remote care were developed inside and outside the physical spaces of health units?
29. Did the e-FH/PHC use remote care in PHC during the critical period of the pandemic (2020-2022.1)? Yes/no

30. Which strategy(ies)? (Teleconsultation, teleconsultancy, telediagnostics, teleregulation, telemonitoring, teleorientation, second formative opinion, other)
31. Are the e-FH/PHC still using remote care in PHC? Yes/no
32. If yes, which strategy(ies)? (Teleconsultation, teleconsultancy, telediagnostics, teleregulation, telemonitoring, teleorientation, second formative opinion, other)
33. If yes, how often (daily, weekly, biweekly, monthly, occasionally)?
34. How many professionals used remote care in PHC during the critical period of the pandemic (2020-2022.1)?
35. How many professionals currently use remote care in PHC?
36. Is there technical assistance service for the digital tools used in remote care in PHC? Yes/no/don't know
37. If yes, preventive or corrective?
38. Has there been adaptation of the physical infrastructure of the Basic Health Unit (UBS) to receive in-person and remote demands? Yes/no
39. If yes, which one(s)? ___________________________
40. Has there been adaptation of the technological infrastructure (computers, internet, tablets, routers, printer, webcam, microphone, speakers, other) of the UBS to receive in-person and remote demands? Yes/no
41. If yes, which one(s)? ___________________________
