# Supplementary material for: Preparation and validation of the instrument “QualiAPS digital—Brazil” for assessing digital health care in primary health care: a required tool
Source: Front Public Health. 2024 Jul 16;12:1304148. doi: 10.3389/fpubh.2024.1304148 (PMC11286592; doi:10.3389/fpubh.2024.1304148)
Supplement: Supplementary file 5 [file Data_Sheet_5.PDF]

VALIDATION OF THE QUALI APS DIGITAL – BRAZIL INSTRUMENT FOR ASSESSING THE QUALITY OF CARE IN DIGITAL HEALTH IN PRIMARY HEALTH CARE

Authors: Renan Cabral de Figueirêdo, Isis de Siqueira Silva, Aguinaldo José de Araújo, Cícera Renata Diniz Vieira Silva, Cláudia Santos Martiniano, Ewerton William Gomes Brito, Pedro Bezerra Xavier, Severina Alice da Costa Uchôa.

MATRIX OF INDICATORS “QUALI APS DIGITAL BRASIL” FINAL VERSION VALIDATED BY EXPERTS IN DIGITAL HEALTH

COMPONENTS		
**STRUCTURE**	**PROCEDURE**	**RESULTS**
DIMENSIONS		
RESOURCES (R): 14 indicators	TECHNIQUE (T): 4 indicators<br>ORGANIZATIONAL (O): 6 indicators<br>RELATIONAL (RE): 3 indicators	SHORT TERM RESULT (ST): 6 indicators<br>MEDIUM TERM RESULT (MT): 4 indicators
INDICATORS		
**Financial resources**<br><br>(R1) Financial resources destined to fund actions in digital health (teleconsultation /teleservice, telemonitoring, virtual home visit, health promotion, disease prevention, among others) in Primary Health Care (quantitative and percentage).	(T1) Individual and collective digital health actions carried out by Family Health/Primary Health Care teams in remote care.<br>(T2) Strategies, programs and projects in digital health developed inside and outside the physical spaces of health units.<br>(T3) Qualification of health professionals and managers for permanent health education through technological resources and aiming at their expansion for use among professionals and users.<br>(T4) Adequate technical support for the use of technologies with a guarantee of security and protection of personal data.	(ST1) Expansion of the care offer with the choice of the most appropriate practices, technologies and instruments to support the lines of care, based on successful cases.<br>(ST2) More accessible health care and maintenance of supply.<br>(ST3) Development and implementation of synchronous, asynchronous and monitoring technological tools, promoting management, assistance and educational actions from the perspective of Primary Health Care.<br>(ST4) Institutionalization of training programs and continuing education with the inclusion of digital health aimed at professionals and users of Primary Health Care.<br>(ST5) Active governance system connected to the needs of the services, incentives for the expansion of digital

		health programs, quality improvement cycles and evaluative research.<br>(ST6) Protagonism of the actors involved: clear definition of responsibilities, trust in technologies, satisfaction of the actors involved and practices within regulatory and ethical standards.
**Collaborators**<br>(R2) Number of managers (Municipal Health Department/ Primary Health Care /Family Health Unit) who implemented some project, program or set of actions in digital health (teleconsultation /teleservice, telemonitoring, virtual home visit, health promotion, disease prevention, among others) in Primary Health Care (quantitative and percentage).<br>(R3) Number of Family Health/Primary Health Care teams that use/used remote care in Primary Health Care.<br>(R4) Number of professionals who use/used remote care in Primary Health Care.<br>(R5) Categories of professionals (health, information technology, management) involved in remote care at the unit, district or central level.<br>(R6) Information and Communication Technology professionals involved/offering support in digital health actions in Primary Health Care (quantitative and health training).	**Careful**<br>(O1) Offer of digital health actions aligned with guarantees of access, integrality and longitudinal care, coordination of care, family and community guidance and cultural competence.<br>(O2) Health surveillance actions through Information and Communication Technologies in territories to support vulnerable groups.<br>**Governance**<br>(O3) Interoperability between information systems (e-Sus and municipal systems) with devices/systems used in remote care.<br>(O4) Planning the feasibility of technological solutions for health demands in Primary Health Care with monitoring and management of resources.<br>(O5) Management incentive actions for innovations in management, care and educational technologies.<br>(O6) Partnerships with the Municipal Health Department for research aimed at digital solutions for health problems in Primary Health Care.	(MT1) Efficacy (capacity to produce improvement) of digital health in the quality of care offered, with positive effects on the adherence of the actors involved, satisfaction/acceptability of health users, resolution, technical accuracy (relevance of technology choices in relation to health problems), continuity and coordination of care in Health Care Networks.<br>(MT2) Expansion of computerization in Primary Health Care: strengthening information systems with data integrity (reliability and consistency of information during its useful life) and operational systems.<br>(MT3) Expansion of the interconnectivity and interlocution of digital technologies to other levels of the Health Care Networks<br>(MT4) Increased economic efficiency (maximization of resources with social well-being) resulting from the adequate allocation of resources to digital health in Primary Health Care in the promotion of equity.
**infrastructure resources**<br>(R7) Adequacy of the physical and technological infrastructure of the health units for the multiple demands (face-to-face and remote).<br>(R 8) Access and quality of equipment and tools for the operation of digital health (computers, notebooks, tablets, wireless routers, external HD, and network servers in health units).<br>(R9) Availability and quality of the internet at the health unit.	(RE1) Professional engagement and community participation in the choice, implementation and evaluation of technologies.<br>(RE2) Reinforcement of welcoming and bonding with users, through the technology interface.<br>(RE3) Use of technology interface with impact on improving teamwork.	-

(R10) Digital tools used: phone calls, videos, text messages, emails, social media, portals, cloud computing, ("cloud" data processing on the internet).<br>(R11) Quality of the information system (e-SUS/ PeC or municipal management systems).<br>(R12) Existence of a technical assistance service for digital tools used in remote care in Primary Health Care.		
**<u>Regulatory/Strategic Resources</u>**<br>(R13) Existence of partnerships (state/municipal telehealth and telemedicine centers; universities; other Information and Communication Technology centers).<br>(R14) Protocols, guidelines and regulations for organizing digital health actions	-	-
DATA COLLECTION TECHNIQUES		
Document analysis (recommendations, informative pages, guidelines, resolutions, laws and ordinances of the Ministry of Health, World Health Organization, Pan American Health Organization, Federal Council of Nursing and Federal Council of Medicine).<br><br>Survey: Intended for professional nurses and physicians from the Family Health/Primary Health Care teams involving the indicators: R2, R3, R5, R7, R8, R9, R10, R11, R14 and T1.<br><br>Interview: - With managers (Health secretaries, Primary Health Care coordinators, health district managers and managers of basic health units) involving the indicators: R1, R2, R3, R4, R5, R12, R13, R14, T3, O4, O5, O6, ST3, ST4, ST5, MT1, MT2, MT3 and MT4.<br>- With Information Technology professionals involving the indicators: R6, R12, T1, T4, O3 and MT2.<br>- With professional nurses and doctors from the Family Health/Primary Health Care teams involving the indicators: T2, T3, O1, O2, O3, O5, RE2, RE3, ST1, ST2 and ST6.<br><br>Focus Group: With Community Health Workers professionals from the Family Health/Primary Health Care teams involving the indicators: R5, R7, R8, R9, R10, R11, R14, T1, RE1, RE2, RE3, ST1, ST2 and ST6.		

Source: Prepared by the research authors (2023).
