# Supplementary material for: Preparation and validation of the instrument “QualiAPS digital—Brazil” for assessing digital health care in primary health care: a required tool
Source: Front Public Health. 2024 Jul 16;12:1304148. doi: 10.3389/fpubh.2024.1304148 (PMC11286592; doi:10.3389/fpubh.2024.1304148)
Supplement: Supplementary file 6 [file Data_Sheet_6.PDF]

# VALIDATION OF THE QUALI APS DIGITAL – BRAZIL INSTRUMENT FOR ASSESSING THE QUALITY OF CARE IN DIGITAL HEALTH IN PRIMARY HEALTH CARE

**Authors: Renan Cabral de Figueirêdo, Isis de Siqueira Silva, Aguinaldo José de Araújo, Cícera Renata Diniz Vieira Silva, Cláudia Santos Martiniano, Ewerton William Gomes Brito, Pedro Bezerra Xavier, Severina Alice da Costa Uchôa.**

## MATRIX OF INDICATORS “QUALI APS DIGITAL – BRAZIL” VERSION 1

| COMPONENTS | | |
|---|---|---|
| **STRUCTURE** | **PROCEDURE** | **RESULTS** |
| DIMENSIONS | | |
| RESOURCES(R): 14 indicators | TECHNIQUE (T): 4 indicators<br>ORGANIZATIONAL (O): 6 indicators<br>RELATIONAL (RE): 3 indicators | SHORT TERM / PRODUCTS (ST): 6 indicators<br>MEDIUM TERM (MT): 4 indicators |
| INDICATORS | | |
| **<u>Financial resources</u>**<br>(R1) Quantitative and percentage of **transfer of financial resources** to the Municipal Health Secretariat (MHS) by the Ministry of Health (MH) or by other public, private or philanthropic entities to fund digital health actions [2] in Primary Health Care (PHC) | (T1) **Offer of individual and collective** digital health actions carried out by professionals from the Family Health Teams (e-FH or e-PHC ) for health maintenance, from promotion, protection, prevention, diagnosis, to treatment, rehabilitation, palliative care and harm reduction, indications (COVID 19 and/or other clinical or epidemiological conditions) categories of professionals involved, target population and frequency (before, and or after the onset of COVID 19, only during the social distancing measures of COVID 19, or with continuity with suspension of these measures.<br>(T2) **Strategies, programs and projects** developed inside and outside the physical spaces of health units<br>(T3) **Offer and qualification of** health professionals and managers for continuing education in health through technological resources and aiming at their expansion for use among professionals and users. | (ST1) **Expansion of the care offer** with the choice of the most appropriate practices, technologies and instruments to support the lines of care, based on successful cases.<br>(ST2) **More accessible and sustainable services**<br>(ST3) **Development and implementation of synchronous, asynchronous and monitoring technological tools**, promoting managerial, assistance and educational actions from the PHC perspective.<br>(ST4) **Institutionalization of training and continuing education programs** with the inclusion of digital health aimed at PHC professionals and users.<br>(ST5) Active **governance system** connected to the needs of the services, incentives for the expansion of digital health programs, quality improvement cycles and evaluative research.<br>(ST6) **Protagonism** of the actors involved: clear definition of responsibilities, trust in technologies, |

| | | |
|---|---|---|
| | (T4) Adequate **technical support for the use of technologies with guaranteed security and protection of personal data** | satisfaction of the actors involved and practices within regulatory and ethical standards. |
| **<u>Collaborators</u>**<br>(R2) Number of managers (MHS/PHC/ Family Health Unit - FHU) who implemented some project, program or set of actions in digital health [3] per FHU.<br>(R3) Quantitative and categories of professionals from the Family Health Teams (e-FH and e-PHC)[4] who develop or have developed digital health actions in the unit/domicile/community.<br>(R4) Number of technicians and/or developers of Information and Communication Technology (ICT[2]) involved in digital health actions[2] in Primary Health Care (PHC)[3] | **<u>Careful</u>**<br>(O1) **Offer of** digital health actions aligned with guarantees of access, integrality and longitudinal care, coordination and ordering of care, family and community guidance and cultural competence[2]<br>(O2) Actions in **Health Surveillance** through ICT[2] in territories to support vulnerable groups<br>**<u>Governance</u>**<br>(O3) **Interoperability** conditions of operating systems [5] such as the interoperability between the Health Information System for Primary Care – HISPC and its applications (Electronic Citizen Record – ECR, e-SUS Território e Atividade Coletiva), and among other systems and applications used by professionals in care.<br>(O4) **Planning the feasibility** of technological solutions for health demands in PHC with monitoring and management of resources.<br>(O5) **Incentive actions** by the Municipal Health Secretariat (MHS) for innovations in management, assistance and educational technologies.<br>(O6) MHS partnerships for **research** aimed at digital solutions for health problems in PHC. | (MT1) Effectiveness (capacity to produce improvement) of digital health[2] in the quality of care offered[7], with positive effects on the adherence of the actors involved ([1.**]) , satisfaction/acceptability of health users[8], resoluteness[7], accuracy technique (pertinence of technology choices in relation to health problems)[7] , continuity and coordination of care in Health Care Networks (HCN)[3]<br>(MT2) **Expansion of computerization in PHC**: strengthening information systems with data integrity (reliability and consistency of information throughout its useful life cycle) and operational systems.<br>(MT3) **Expansion, interconnectivity and intersectoral dialogue of digital technologies to other levels of the Health Care Networks**<br>(MT4) **Increased economic efficiency** (maximization of resources with social well-being) resulting from the adequate allocation of resources to digital health in PHC in the promotion of equity. |
| **<u>infrastructure resources</u>**<br>(R5) Number of Family Health Units (FHU) that carry out or have carried out Digital Health actions[2]<br>(R6) Geographical accessibility (possibility of access) and adequacy of the physical spaces of the FHU - Family Health Units) for multiple demands, face-to-face/remote COVID19 and non-COVID19.<br>(R7) Access and quality of equipment and tools for the operation of digital health (computers, notebooks, tablets, wireless routers, external HD, and network servers in the FHU. | (RE1) Professional **engagement and community participation** in the choice, implementation and evaluation of technologies.<br>(RE2) **Reinforcement of welcoming** and **bonding** with users, even with the technology interface<br>(RE3) **Creativity and willingness** of managers and professionals to improve teamwork. | - |

| | | |
|---|---|---|
| (R8) Availability and quality of the internet, connectivity (computers with access to the internet network), and integration between systems (internet of things such as sensors, smart monitoring that can be viewed on multiple devices at the same time)<br>(R9) Digital tools (ICT)[2] used: phone calls, videos, text messages through apps, social media, portals, cloud computing, ("cloud" data processing on the internet)<br>(R10) Quality of information systems, data management and interface with technology users (health professionals and users)<br>(R11) Existence of provision of technical assistance to digital services provided by Family Health Units. | | |
| **Regulatory/Strategic Resources**<br>(R12) Partnerships (state/municipal telehealth centers; universities; other ICT centers[2].<br>(R13) Information System including actions in digital health<br>(R14) Existence and adequacy of protocols, guidelines and regulations for the organization of digital health actions. | - | - |
| DATA COLLECTION TECHNIQUES | | |
| Document analysis<br><br>Survey: Managers and Professionals (doctor e-FH and e-PHC and IT) | Survey: Managers and Professionals (doctor e-FH and e-PHC and IT)<br><br>Interview: Professionals (e-FH and e-PHC doctors)<br>e-FH Community Health Agents)<br><br>Focal Group: Professionals (nurses and Community Health Workers e-FH) | Survey: Managers and Professionals (doctor e-FH and e-PHC and IT)<br><br>Interview: Professionals (e-FH and e-PHC doctors)<br>e-FH Community Health Workers)<br><br>Focal Group: Professionals (nurses and Community Health Workers e-FH) |
