# Supplementary material for: Preparation and validation of the instrument “QualiAPS digital—Brazil” for assessing digital health care in primary health care: a required tool
Source: Front Public Health. 2024 Jul 16;12:1304148. doi: 10.3389/fpubh.2024.1304148 (PMC11286592; doi:10.3389/fpubh.2024.1304148)
Supplement: Supplementary file 7 [file Data_Sheet_7.PDF]

SILVA, Cícera Renata Diniz Vieira; DA COSTA UCHOA, Severina Alice (2022). Model for Assessing Digital Health in PHC.

Structure

Resources

Collaborators

- Health professionals (Family Health Teams)
- Managers (Health Department, Primary Care coordination)
- Technical professionals in ICT's

Financial resources

- Finances to fund digital health strategies in PHC.

Infrastructure Resources

- Health units (physical space);
-Equipment, servers and tools (Internet connection, cloud computing, Web systems, Desktop, mobile applications, electronic devices connected to the internet, portals, electronic records, computers, notebooks, telephones, cell phones, etc.).

Normative/strategic resources

- Protocols, guidelines and regulations;
- Data / datasets;
- Partnership network.

Procedure

Activities

USE OF DIGITAL HEALTH STRATEGIES TO OPERATIONALIZE THE FOLLOWING DIMENSIONS:

TECHNICAL DIMENSION

- Individual and collective actions to maintain health, from promotion, prevention, protection, diagnosis, treatment, rehabilitation, harm reduction, palliative care.
- Strategies, programs and projects developed inside and outside the physical spaces of health units.
- Qualification of health professionals and managers through technological resources and aiming for their expansion for use among professionals and users.
- Adequate technical support for the use of technologies that guarantee security and protection of personal data.

ORGANIZATIONAL DIMENSION

- Care: Offering actions aligned with guaranteeing access to care, comprehensiveness and longitudinality of care, coordination and ordering of care, taking into account the literacy, culture and values of users/community. Health surveillance in territories to support vulnerable groups.
- Governance: 1) Interoperability between systems; 2) Planning the feasibility of technological solutions for PHC demands; 3) Actions to encourage innovations in management, assistance and educational technologies, with a view to adapting work processes; 4) Management and monitoring of the use of resources for digital health; 5) Partnerships with academia and companies to acquire solutions and/or develop research and/or development projects.

RELATIONAL DIMENSION

- Professional engagement and community participation in the choice, implementation and evaluation of technologies.
- Reinforcing reception and strengthening the bond with users, even with the technology interface.
- Creativity and willingness to improve teamwork.

Results

Products

Expanding the provision of care by choosing the most appropriate practices, technologies and instruments to support lines of care, based on success stories in digital PHC.

Development and implementation of synchronous, asynchronous and monitoring technological tools, promoting management, assistance and educational actions from the perspective of PHC.

Training and continuing education programs with digital health strategies for PHC professionals and users.

Active governance system connected to the needs of services, with incentives for expanding programs and initiatives in digital health, improving quality and evaluating research.

Protagonism of the actors involved in the care process, with clear definition of responsibilities.

Intermediate results

Access to appropriate tools for the integrated development of digital health in PHC and reaching target populations. More accessible and sustainable services.

Efficacy of digital health in the quality of care offered, with positive effects on adherence, satisfaction, acceptability, resolution, technical accuracy, continuity and coordination of care.

Leaders and professionals trained to use digital strategies. Work process adapted to increase benefits and minimize barriers, as well as increase professionals' confidence and security with its use.

Expansion of computerization of spaces and strengthening of information systems (data integrity) and operational systems.

Interconnectivity and intersectoral dialogue with expansion of technologies to other care scenarios and articulated with PHC, in care networks.

Increased economic efficiency and adequate allocation of resources for digital health in PHC.

Strengthening collaboration and communication between actors (users, professionals and managers).

Impacts

- Potential and sustainable use of management, care and educational health technologies in PHC.
- Institutionalization of evaluation for continuous improvement of the quality of digital health in PHC.
- Digital health providing PHC that is more effective and closer to users.
